# *Arabidopsis* mutant *sk156* reveals complex regulation of *SPL15* in a *miR156*-controlled gene network

**DOI:** 10.1186/1471-2229-12-169

**Published:** 2012-09-18

**Authors:** Shu Wei, Margaret Y Gruber, Bianyun Yu, Ming-Jun Gao, George G Khachatourians, Dwayne D Hegedus, Isobel AP Parkin, Abdelali Hannoufa

**Affiliations:** 1 College of Tea & Food Science and Technology, Anhui Agricultural University, 130 Changjiang Blvd West, Hefei, 230036, China; 2 Agriculture and Agri-Food Canada, 107 Science Place, Saskatoon, SK, S7N 0X2, Canada; 3 Current address: Plant Biotechnology Institute, National Research Council of Canada, 110 Gymnasium Place, Saskatoon, SK, S7N 0W9, Canada; 4 Department of Food and Bioproduct Sciences, University of Saskatchewan, 51 Campus Drive, Saskatoon, SK, S7N 5A8, Canada; 5 Agriculture and Agri-Food Canada, 1391 Sandford Street, London, ON, N5V 5T3, Canada

## Abstract

**Background:**

The *Arabidopsis microRNA156* (*miR156*) regulates 11 members of the *SQUAMOSA PROMOTER BINDING PROTEIN LIKE* (*SPL*) family by base pairing to complementary target mRNAs. Each *SPL* gene further regulates a set of other genes; thus, miR156 controls numerous genes through a complex gene regulation network. Increased axillary branching occurs in transgenic *Arabidopsis* overexpressing *miR156b*, similar to that observed in loss-of-function *max3* and *max4* mutants with lesions in carotenoid cleavage dioxygenases. *Arabidopsis miR156b* was found to enhance carotenoid levels and reproductive shoot branching when expressed in *Brassica napus,* suggesting a link between *miR156b* expression and carotenoid metabolism. However, details of the miR156 regulatory network of *SPL* genes related to carotenoid metabolism are not known.

**Results:**

In this study, an *Arabidopsis* T-DNA enhancer mutant, *sk156*, was identified due to its altered branching and trichome morphology and increased seed carotenoid levels compared to wild type (WT) ecovar Columbia. Enhanced *miR156b* expression due to the 35S enhancers present on the T-DNA insert was responsible for these phenotypes. Constitutive and leaf primodium-specific expression of a miR156-insensitive (mutated) *SPL15* (*SPL15m*) largely restored WT seed carotenoid levels and plant morphology when expressed in *sk156*. The *Arabidopsis* native miR156-sensitive *SPL15* (*SPL15n*) and *SPL15m* driven by a native *SPL15* promoter did not restore the WT phenotype in *sk156*. Our findings suggest that *SPL15* function is somewhat redundant with other SPL family members, which collectively affect plant phenotypes. Moreover, substantially decreased *miR156b* transcript levels in *sk156* expressing *SPL15m,* together with the presence of multiple repeats of SPL-binding GTAC core sequence close to the *miR156b* transcription start site, suggested feedback regulation of *miR156b* expression by SPL15. This was supported by the demonstration of specific *in vitro* interaction between DNA-binding SBP domain of SPL15 and the proximal promoter sequence of *miR156b.*

**Conclusions:**

Enhanced *miR156b* expression in *sk156* leads to the mutant phenotype including carotenoid levels in the seed through suppression of *SPL15* and other SPL target genes. Moreover, SPL15 has a regulatory role not only for downstream components, but also for its own upstream regulator *miR156b*.

## Background

The *Arabidopsis* miR156 family has eight members and is highly conserved in the plant kingdom. It has been identified in 45 plant species [1]. MiR156 is known to repress *SPL* (*SQUAMOSA PROMOTER BINDING PROTEIN-LIKE*) genes [2-4], which are plant-specific transcription factors containing the SBP (SQUAMOSA promoter binding protein) box [5]. In *Arabidopsis*, 11 *SPL* genes are targeted by miR156 [2,4,6,7], and *in silico* full genome analysis showed that no other genes in *Arabidopsis* have the segment complimentary to miR156 [2]. Some details of the relationship between the miR156 regulatory network downstream of *SPL* genes and flowering enhancement and flavonoid metabolism have been revealed [8-10]. But this is clearly an area of research that still needs strong attention.

Diverse and redundant roles of some individual *SPL* genes in plant morphology and development have been reported. The *SPL* genes targeted by miR156 can be grouped into four major clades: *SPL3/SPL4/SPL5*, *SPL2/SPL10/SPL11*, *SPL9/SPL15*, and *SPL6/SPL13*[7]. *SPL3, SPL4* and *SPL5* exhibit partially redundant effects on plant juvenile-to-adult transition [4,11,12]. *SPL9* and *SPL3* directly activate *MADS* box genes that promote flowering [8]. In addition, *SPL9* and *SPL15* interchangeably control shoot maturation and leaf initiation [13]. *SPL10* and *SPL9* expression in leaf primordia modulated by miR156 affects initiation of new leaves at the shoot apical meristem [14]. *SPL2, SPL10* and *SPL11* were each able to control leaf lamina shape in association with shoot maturation in the reproductive phase [15]. Moreover, *SPL* genes (represented by *SPL9*) regulate trichome development via direct interaction with miR172 [9] and the MYB transcription factor genes *TRICHOMELESS1* (*TCL1*) and *TRIPTYCHON* (*TRY*) [16].

Increased axillary branching occurs in transgenic *Arabidopsis* lines expressing *miR156b* under the control of a cauliflower mosaic virus 35S promoter (CaMV35S) [6], similar to that observed in loss-of-function *max3* and *max4* mutants with lesions in carotenoid cleavage dioxygenases, CCD7 and CCD8, respectively [17-19]. These *max* mutations cause defective biosynthesis of strigolactones; a group of carotenoid-derived hormones that inhibit shoot branching [20,21]. The morphological similarities between these transgenic and mutant lines suggest a link between *miR156b* expression and carotenoid metabolism. This was partially confirmed when *Arabidopsis miR156b* was found to enhance carotenoid levels and reproductive shoot branching when expressed in *Brassica napus*[22].

In this study, we report on a new *Arabidopsis* activation tagged mutant, *sk156*, with strongly enhanced expression of *miR156b.* MiR156b-induced *SPL15* suppression was partially responsible for the increased seed carotenoid abundance and altered plant morphology observed in *sk156*. In addition, we highlight a new SPL15 feedback loop which controls expression of *miR156b*, likely via a physical interaction between the SPL15 SBP domain and the promoter of *miR156b*.

## Results

### Phenotypes of the *sk156* mutant

**Figure 1:**
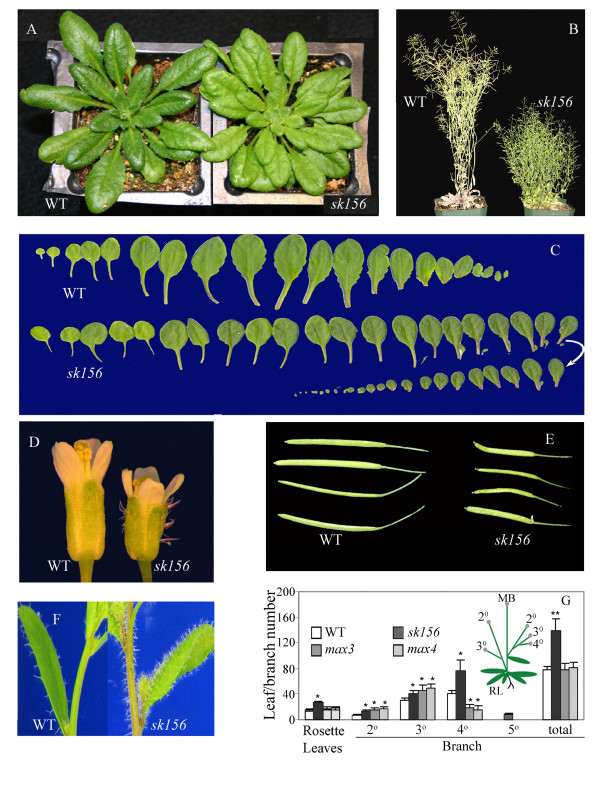
**Morphological phenotype of the Arabidopsis *sk156 *mutant.** (**A**) 30-d-old rosette plants. (**B**) 8-week-old mature plants. (**C**) 30-d-old plant leaves. WT on top, *sk156* on bottom. (**D**) Flowers, showing trichome-bearing *sk156* sepals (right). (**E**) Siliques at 14 days post-anthesis. (**F**) Trichome enhancement on cauline stems and leaves of *sk156*. (**G**) Enhanced rosette leaf and branch numbers (insert defines branch order). Data were the mean of thirty 8-week-old plants. MB, main stem; RL, rosette leaves. A Duncan's multiple range test showed significant differences of the means (± standard deviation) between mutant lines and WT at p < 0.01 (**) and p < 0.05 (*).

The *sk156* mutant was selected from an *Arabidopsis* activation-tagged mutant population that was developed using a T-DNA construct containing four CaMV35S enhancers [23]. Compared to the parental wild type (WT) Col-4, *sk156* exhibited the following morphological changes: increased numbers of rosette leaves which were slightly pale, smaller and rounder, increased reproductive branching, ectopic expression of trichomes on flower sepals and shoot tips, delayed bolting, severely stunted cauline stems, and decreased flower and silique sizes (Figure 1).

For comparison, we examined the phenotypes of *max3-9*[17] and *max4-1*[19] mutants, which had been confirmed previously to contain defective *CCD7* and *CCD8* genes, respectively (Additional file 1). In our hands, these *max* mutants showed increased secondary and tertiary branches (Figure 1G), which was consistent with an earlier report that *max* mutants showed increased inflorescence number compared to WT plants [24]. However the cauline stems of these two *max* mutants were not significantly stunted as in *sk156*. The branching pattern of *sk156* was also somewhat distinct compared with *max* mutants; *sk156* had more quaternary and quinary branches and the *max* mutants mainly had increased secondary and tertiary branches.

**Figure 2:**
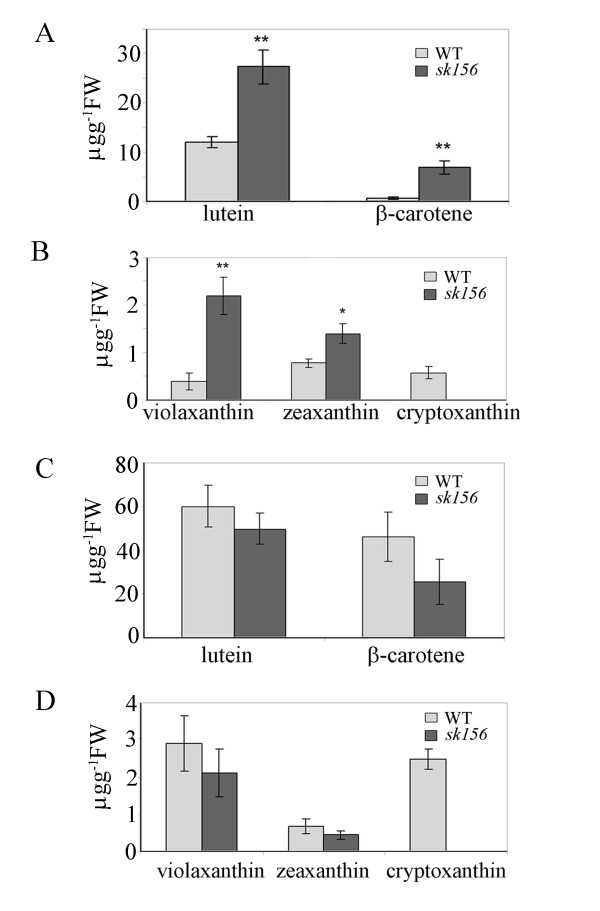
**Carotenoid levels in *sk156 *mutant.** (**A**) and (**B**) Carotenoid levels in mature seeds. (**C**) and (**D**) Carotenoid levels in leaves of 30-d-old plants. A Duncan's multiple range test showed significant differences of the means (±standard deviation) between mutant lines and WT at p < 0.01 (**) and p < 0.05 (*).

The abundance of five major carotenoid compounds was examined in the mature seeds and leaves of *sk156* and WT plants. Levels of lutein, β-carotene, violaxanthin, and zeaxanthin were 2.3-, 11.7, 5.5-, and 1.8- fold higher in *sk156* seeds than in WT seeds (Figure 2A and B). Slight decreases (but not statistically significant) in the levels of major carotenoids in leaves were found in *sk156* compared to WT (Figure 2C and D). Cryptoxanthin was undetectable in either tissue of the mutant, but present at 0.62 μg g^-1^ FW (fresh weight) in WT seeds and 4-fold higher in WT leaves.

### Molecular analysis of *sk156*

**Figure 3:**
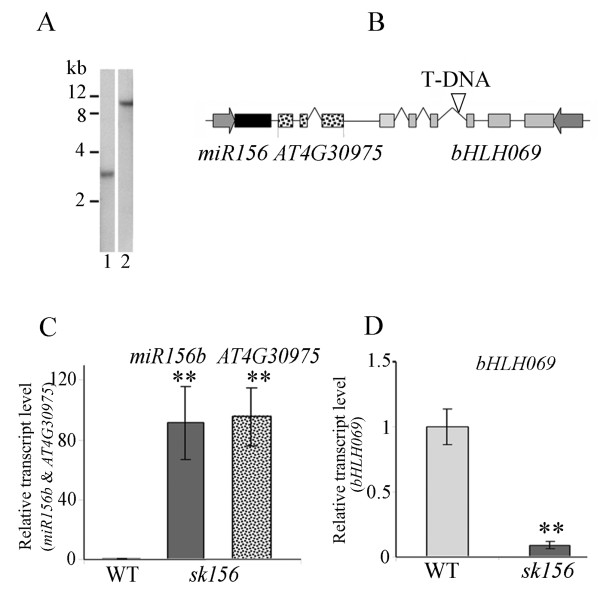
**T-DNA insertion site and affected genes in *sk156 *mutant.** (**A**) Southern blot analysis of *sk156* showing a single T-DNA insertion. Lane 1, digested with *Bam*HI. Lane 2, digested with *Hind*III. (**B**) Schematic of T-DNA inserted in the third intron of *bHLH069* shown by ‘∇’ (empty triangle). Boxes, exons; Lines, introns and intergenic regions. Thick grey arrows show promoter regions and their transcription direction. (**C**) Transcript levels for *miR156b* and unknown RNA gene (AT4G30975) in *sk156* relative to WT (set at 1) using qPCR. (**D**) Transcript levels for *bHLH069* in *sk156* relative to WT (set at 1) using qPCR. A Duncan's multiple range test in (**C**) and (**D**) showed significant differences of the means (± standard deviation) between *sk156* and WT Col-4 at p < 0.01 (**) and p < 0.05 (*).

Segregation analysis of heterozygous *sk156* mutant offspring revealed that the ratio of resistant-to-sensitive seedlings (271:109, respectively) was well within the expected 3:1 ratio (χ^2^, p = 0.05), indicating that there was a single T-DNA insertion site. This was confirmed by Southern blot analysis (Figure 3A). Sequencing of the T-DNA-flanking regions indicated that the T-DNA was located in AT4G30980 (Figure 3B), which encodes a basic helix-loop-helix (bHLH) family protein bHLH069 [25,26]. CaMV 35S enhancers in the T-DNA were within close proximity to five other gene loci: AT4G30975 (unknown RNA gene), AT4G30972 (*miR156b*), AT4G30970 (unknown protein) and AT4g30960 (SOS2-like protein kinase PKS4).

The *sk156* phenotypes were dominant when crossed with WT, suggesting that the altered phenotypes in *sk156* were tightly linked to enhanced expression of gene(s) adjacent to the T-DNA, rather than gene disruption. Thus, transcript levels of genes close to the T-DNA insertion site were compared in the mutant and WT plants using quantitative RT-PCR. Transcript levels of AT4G30972 (*miR156b*) and the unknown RNA gene AT4G30975 were increased 91.4-fold (± 25.8) and 95.8-fold (±18.6), respectively, in *sk156* (Figure 3C), while the T-DNA-disrupted AT4*g*30980 (*bHLH069*) was repressed 11.1-fold (Figure 3D). No significant changes in transcript levels were detected for the other adjacent genes (data not shown). This indicated that the four CaVM 35S enhancers inserted into *bHLH069* caused the activation of *miR156b* and the unknown RNA gene.

**Figure 4:**
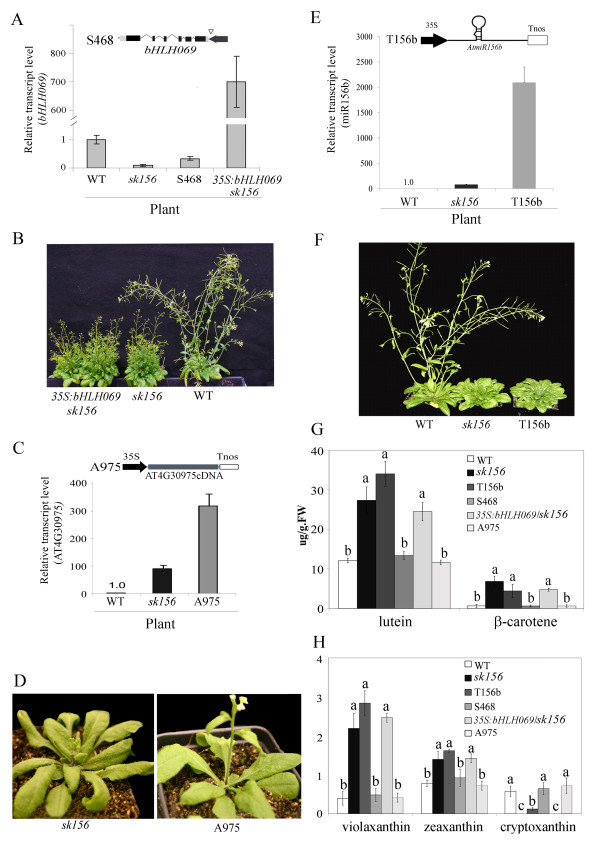
**Involvement of *miR156b*, *bHLH069*, and the unknown RNA *AT4G30975 *in the phenotypes of the *sk156 *mutant.** (**A**) Transcript levels of *bHLH069* in WT (set at 1), *sk156*, mutant line S468 (SALK_032468), and in *sk156* transformants complemented with a *35S:bHLH069* construct relative to WT (set at 1). Insert, schematic of T-DNA insertion site (triangle) in *bHLH069* (*AT4G30980*) in S468 shows above. Exons (black boxes), untranslated regions (light grey boxes), promoter direction (dark arrow). (**B**) Morphology of 45-d-old transgenic *sk156* complemented with *35S:bHLH069*. *Sk156*, and WT plants are also shown. (**C**) Transcript levels of unknown RNA gene *AT4G30975* in WT (set at 1), *sk156*, and *35S:AT4G30975cDNA*-complemented *sk156* (A975) plants. Schematic of AT4G30975 expression construct shows above. (**D**) Morphology of 35-d-old transgenic plant A975 compared to *sk156*. (**E**) Transcript levels of miR156 in WT, *sk156*, and *35S:miR156b*-complemented *sk156* (T156b) plants. Schematic of *35S:miR156b* expression construct shows above. (**F**) Morphology of 40-d-old transgenic plants from *sk156* and *35S:miR156b*-over-expressing T156b lines compared to WT. (**G**) and (**H**) Seed carotenoid levels in *sk156*, T156b, A975, S468, *35S:bHLH069*/*sk156*, and WT plants. A Duncan's multiple range test showed significant differences of the means (± standard deviation) compared to WT at p < 0.05. Means containing the same letter for the same compounds are not significantly different.

To determine which of the three T-DNA affected genes was responsible for the altered phenotypes in *sk156*, including enhanced seed carotenoid levels, four types of plants were obtained and examined (Table 1). These included a *bHLH069* knockout mutant S468 (SALK_032468) (Figure 4A), transgenic *sk156* over-expressing a *35S*:*bHLH069* cDNA (Figure 4B) transgenic WT carrying *35S:AT4G30975* cassette (A975, the unknown RNA gene) (Figure 4C and D), and transgenic *Arabidopsis* WT carrying a *35S*:*miR156b* cassette (T156b) (Figure 4E and F) [12]. Transcript analysis showed that expression of *bHLH069* was defective in both S468 and *sk156* but enhanced in *sk156* over-expressing *bHLH096* (Figure 4A). Morphology was indistinguishable between *sk156* and *sk156* over-expressing *bHLH096* (Figure 4B) and between the SALK mutant and WT (data not shown). Additionally, over-expression of the unknown RNA cDNA in WT (line A975) (Figure 4C) did not produce the *sk156* mutant phenotype (Figure 4D). However, the 35S promoter-driven *miR156b* overexpression resulted in morphological characteristics in line T156b similar to those of *sk156* (Figure 4F).

**Table 1:** **Summary of transgenic and mutant *Arabidopsis *lines characterized in this study**

**Plant Lines**	**Construct, key element and genetic background**	**Gene expression vs. WT control**	**Key phenotypes**
*sk156*	pSKI015, 35S enhancer, Col-4 background	91.4- and 95.8-fold higher expression for *miR156b*, At4g30975	Increased seed carotenoids and branching
S468	pROK2, SALK_032468, Col-0 background	11.1 fold less expression for AT4G30980	Similar to WT
*35S:bHLH069*	Modified pBI121, 35S:At4g30980 cDNA, *sk156* background	708.4 fold higher expression for bHLH069	Similar to *sk156*
T156b	Modified pBI121, 35S:*miR156b*, Col-4 background	2134.6 fold higher expression for *miR156b*	Similar to *sk156*
A975	Modified pBI121, 35S:At4g30975 cDNA, Col-4 background	332.5 fold higher expression for At4g30975	Similar to WT
CS2117	pDs-Lox, CS852117, Col background	4.3 fold less expression for *SPL15*	Slightly stunted cauline stem and increased seed carotenoids.
Col background	4.3 fold less expression for *SPL15*	Slightly stunted cauline stem and increased seed carotenoids.	
CS6815	pDs-Lox, CS856815, Col background	5.2 fold less expression for *SPL15*	Similar to CS2117.
S8712	pROK2, SALK_138712, Columbia background	4.7 fold less expression for *SPL15*	Similar to CS2117.
*35S:SPL15n*	Modified pBI121,*35S:SPL15n* (miR156 sensitive), *sk156* background	4.7 fold less expresson for *SPL15*.	Similar to *sk156*
*35S: SPL15m*	Modified pBI121, *35S:SPL15m* (miR156 insensitive), *sk156* background	22.8-613.6 fold higher expression of SPL15	Similar to WT, changed leaf shape.
*AS1:SPL15n*	Modified pBI121, *AS1:SPL15n* (miR156 sensitive), *sk156* background	3.2 fold less expression for SPL15	Similar to *sk156*, slightly changed leaf color
*AS1:SPL15m*	Modified pBI121,*AS1:SPL15m* (miR156 insensitive), *sk156* background	4.6 fold higher expression for SPL15	Similar to WT, changed leaf shape
*SPL15:SPL15m*	Modified pBI121, *SPL15:SPL15m* (miR156 insensitive), *sk156* background	1.6 fold higher expression for SPL15	Similar to *sk156*, changed leaf shape

Carotenoid analysis further confirmed the role of enhanced *miR156b* expression in generating the *sk156* phenotypes (Figure 4G and H). No significant differences in seed carotenoid abundance were found between the defective *bHLH069* mutant S468 and WT (p ≤ 0.05). Complementation of *sk156* with *bHLH069* did not restore *sk156* seed carotenoid levels back to WT levels, nor did over-expression of the unknown RNA in WT result in enhanced seed carotenoid levels. Only in line T156b overexpressing *miR156b* were seed carotenoid levels raised to levels found in *sk156* seeds (Figure 4G and H). These data clearly indicate a role for miR156b in the phenotypes of *sk156*.

Due to similar enhanced transcript levels of *miR156b* and AT4G30975 in the *sk156* mutant, yet unchanged morphology and seed carotenoid accumulation in the *AT4G30975* over-expression lines (line A795, Table 1), we examined the relationship between *miR156b* transcripts and *AT4G30975* cDNA. These two transcripts are separated by a 17 bp intergenic fragment (TAIR, version 9). Sequencing of cDNA obtained from the leaves of 4-week-old WT plants revealed a *miR156b* transcript which included both the 17 bp fragment and the full segment of the AT4G30975 transcript (sequencing data not shown). These data show that enhanced expression of *miR156* is the reason for the phenotypic changes in the *sk156* mutant line, regardless of *AT4G30975* co-transcription.

### Suppression of *SPL15* is involved in the *sk156* phenotype

**Figure 5:**
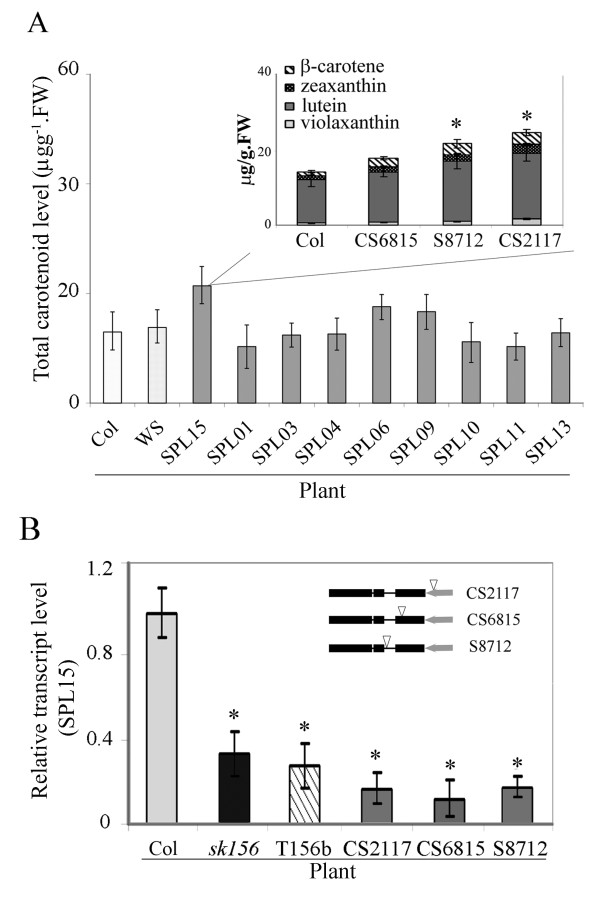
**Involvement of *SPL15 *in phenotypic alteration of *sk156*.** (**A**) Carotenoid levels in *SPL* loss-of-function mutants specifying nine SPL genes, including SPL15. *SPL15* SALK lines in insert show details on specific carotenoid changes. (**B**) *SPL15* transcript levels in *sk156*, *SPL15*-related Salk lines, and T156b relative to WT (Col, set at 1). Insert shows schematic diagrams of T-DNA insertion sites ‘∇’ in *SPL15* SALK mutants CS2117, CS6815 and S8712.Duncan's multiple range test showed significant differences (*) of the means (± standard deviation) between *sk156* and WT (Col or WS) at p < 0.05.

**Figure 6:**
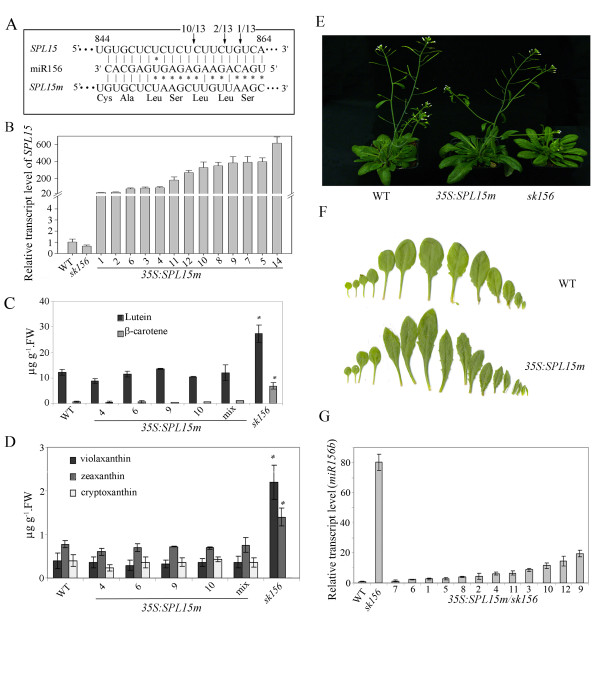
**Expression of mutated *SPL15m *in *sk156 *results restores a WT phenotype and down regulates *miR156b *transcription.** (**A**) Complementarity of *miR156* with *SPL15* sequences. Arrows show three cleavage sites (one used 10 out of 13 times) in *SPL15* mRNA due to interaction with *miR156b*. *SPL15m* shows 11 nucleotides mutated in DNA sequence but unchanged in amino acid sequence. (**B**) *SPL15* transcript levels in independent transgenic *sk156* lines carrying *35S:SPL15m* compared to WT (set at 1) and *sk156*. Error bars are standard deviations of the means. (**C**) and (**D**) Restoration of WT-like carotenoid levels in dry seeds of transgenic *sk156* expressing *SPL15m*. Cryptoxanthin was undetectable in the mutant. (**E**) Restoration of WT-like plant growth in 35-d-old plants of *sk156* expressing *SPL15m*. (**F**) Distinctive elongated leaf morphology of *SPL15m*-transformed plants compared to WT plants. (**G**) *miR156b* transcript levels in different transgenic *sk156* lines expressing *SPL15m* compared to WT (set at 1) and *sk156*. A Duncan's multiple range test was conducted to separate significantly different means. Panels **B**, **G**, ± standard deviation; Panels **C**, **D**, ± standard error; p < 0.01 (**) and p < 0.05 (*).

*Arabidopsis* Columbia has 11 *SPL* genes (two loci for *SPL13*) that are regulated by miR156 [7,9]. To determine which *SPL* geneswere most likely involved in altered carotenoid profiles of *sk156* seeds, mutant lines with confirmed T-DNA insertion knockouts for all miR156-target *SPL* genes (except *SPL5* where mutants were unavailable) were obtained (Additional file 2). The mutant lines were compared for morphology and carotenoid differences with their corresponding ecotype Columbia and Wassilevskija controls. While levels of carotenoids were modestly increased in three *spl15* mutant lines (Figure 5A insert), they were less affected in the remaining *spl* mutants (Figure 5A). Even in the *spl15* mutant lines, the enhanced total carotenoid levels (21.5 ± 1.7μg/g.FW) (Figure 5 insert) were not as high as the sum of each individual carotenoid compound observed in *sk156* (37.8 ± 5.5μg/g.FW) (Figure 6C and D).

The three different *SPL15* “knockout” mutants, CS852117, CS856815 and SALK_138712 (Figure 5A insert) all exhibited reduced *SPL15* transcript levels similar to levels found in the *sk156* mutant and the *miR156b* over-expression line T156b (Figure 5B; Table 1). However, other *spl* mutants had normal cauline stem lengths (data not shown), while *spl15* mutants had slightly stunted stems (Additional file 3) as reported previously [13]. Assays using rapid amplification of 5' complementary DNA ends (5'-RACE) indicated that three cleavage sites exist within the region of the native *SPL15* transcript that is complementary to the miR156 mature sequence, with one site being used more frequently than the other two (Figure 6A). Collectively, these results suggest that *SPL15* is a direct target of miR156 and that it plays a role in the morphology and seed carotenoid phenotypes found in *sk156* mutant line.

### Expression of miR156*-*insensitive *SPL15m* affects the phenotype of *sk156*

Since *spl15* mutants had several phenotypes similar to those of *sk156*, two different *SPL15-*complemented *sk156* lines were developed to determine whether the WT phenotype could be restored in a miR156b-enhanced *sk156* background (Table 1). The first transgenic *sk156* carried a *35S:SPL15n* (native *SPL15*) cassette to avoid any endogenous transcriptional regulation which might be present if using a native *SPL15* promoter (Figure 6A). The second carried *35S:* (mutated *SPL15*), which contains 11 mutated sites within the *SPL15* segment complementary to mature miR156 so that *SPL15m* becomes insensitive to miR156 regulation (Figure 6A). PCR analysis using gene-specific primers confirmed both pSKI015 T-DNA and either *SPL15n* or *SPL15m* within these transgenic lines (Additional file 4). Sequencing of these PCR products confirmed that all *SPL15m*-expressing lines had a *SPL15*-mutated segment (Figure 6A) as expected. *SPL15* transcript levels (sum of *SPL15m* and endogenous *SPL15*) within the *35S:SPL15m*^*+*^ lines were increased by 86.2-fold to 617.4-fold compared to WT (Figure 6B). No significant differences were found in the amount of lutein, vialoxanthin, zeaxanthin and β-carotene in seeds between these *35S*:SPL15*m*-transformed plants and wild type control plants (Figure 6C and D). The transgenic plants showed a largely restored WT morphology in leaf and branch numbers, bolting time, cauline stem length, flower and silique size, and trichome number in reproductive shoots and tissues (Figure 6E). Only leaf morphology was different between transgenic and non-transgenic lines (Figure 6F). This contrasted with miR156-controlled *35S*:*SPL15n*^*+*^ lines (Table 1), which displayed *sk156* phenotypes.

**Figure 7:**
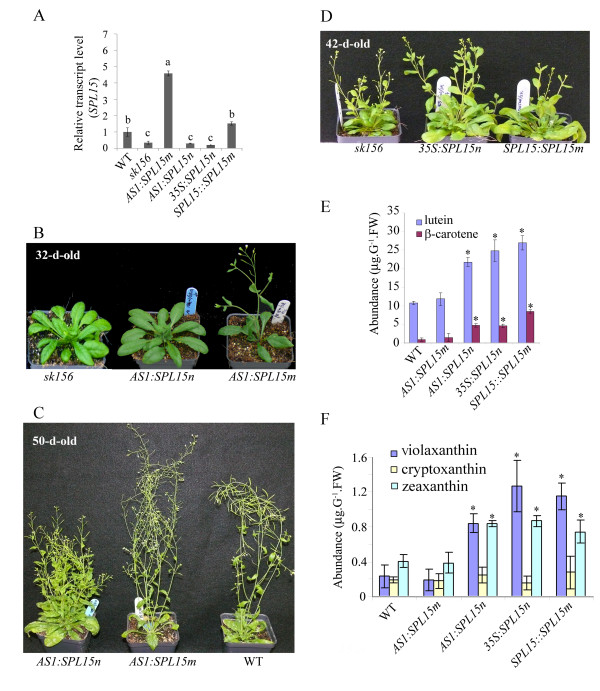
**Leaf primodium-dependent miR156-insensitive *SPL15m *restores WT phenotypes when expressed in *sk156*.** The *sk156* background was transformed with native miR156-sensitive *SPL15n* or miR156-insensitive *SPL15m* expression cassettes each controlled by either an AS1 or 35S promoter. (**A**) *SPL15* transcript levels by qPCR (± standard deviation) in transgenic *sk156* plants complemented with *AS1:SPL15m*, *AS1:SPL15n*, 35S:*SPL15n* or *SPL15:SPL15m* compared to WT (set at 1) and *sk156* (p < 0.05). (**B**) and (**C**) Morphology at bolting (32 d) and maturity (50 d) for plant expressing *SPL15n* or *SPL15m* under the control of AS1 promoter compared to *sk156*. (**D**) Morphology of 42 d flowering lines for *sk156* plant carrying *35S:SPL15n* or *SPL15:SPL15m* compared to *sk156*. (**E**) and (**F**) Carotenoid levels (± standard error) in seeds of *sk156* complemented with *AS1:SPL15m, AS1:SPL15n***,***35S:SPL15n* or *SPL15:SPL15m* compared to those of WT and *sk156*. Panels **A**, **E**, **F**: A Duncan's multiple range test was conducted to separate significantly different means for more than 10 independent transgenic lines (each measured with triplicated seed batches) relative to WT plants at p < 0.05 (*).

Since constitutive expression of *SPL15m* predominantly restored *sk156* phenotypes, native *SPL15n* and mutated *SPL15m* under the control of the young leaf primordia promoter from the *ASYMMETRIC LEAVES 1* (*AS1*) gene [27] were used to generate two additional complemented sets of *sk156* lines. This was to find out whether SPL15 functions at the leaf primordia within the shoot apex as occurs with certain SPL genes controlling plastochron length [14]. PCR analyses using promoter-specific and gene-specific primers (Additional file 5, see materials and methods) confirmed the presence of the transgene in these transgenic plants (Additional file 6). *SPL15m* transcript levels were increased in *AS1* promoter-driven *SPL15m* transgenic *sk156* lines, but not in *AS1* promoter-*SPL15n* lines, compared to WT and *sk156* mutants (Figure 7A). As with *SPL15m* expression directed by the *35S* promoter, expression of *SPL15m* under the control of the *AS1* promoter in the *sk156* background restored WT morphology at different growth stages, whereas *AS1:SPL15n* did not (Figure 7B, C, and D). Moreover, the levels of seed carotenoids in these *ASI:SPL15m* plants were not significantly different from those in WT (p > 0.05), while seed carotenoid abundance was significantly higher in plants carrying the *AS1:SPL15n* cassette (p > 0.05) (Figure 7E and F). These changes in the phenotype of transgenic *sk156* plants expressing the miR156*-*insensitive *AS1:SPL15m* suggested that *SPL15m* could function independently of miR156b and that expression of *SPL15* is effective at the leaf primordia within the shoot apex.

Since the native *SPL15n* appeared to be ineffective at restoring the WT phenotype in the enhanced *miR156b* environment of *sk156*, another set of complemented *sk156* lines was generated to express the miR156*-*insensitive *SPL15m* under the control of the native *SPL15* promoter (Table 1). Morphological traits and carotenoid levels remained unchanged for these transgenic *SPL15:SPL15m* plants compared to uncomplemented *sk156*, except that the leaf shape was similarly elongated as were *35S:SPL15m* plants compared to WT plants (Figure 6F). These data suggested that overwhelming miR156b levels within the *sk156* mutant negatively regulated the expression of the *SPL15n* under its native promoter and prevented restoration of WT seed carotenoid abundance and plant morphology. Moreover, the inability of *SPL15:SPL15m* plants to restore most of the WT phenotype as did *35S:SPL15m*^*+*^ lines suggested that the enhanced seed carotenoid level and morphological traits observed in the *sk156* mutant were the collective result of miR156 suppression of at least some other *SPL* genes rather than *SPL15* alone and that SPL15 functions redundantly with some other *SPL* gene products.

### Transcription of *miR156b* is affected by over-expression of *SPL15m*

Wu and Poethig [12] reported earlier that expression of miR156-insensitive *SPL3* under the control of the CaMV35S promoter causes reduced levels of *miR156* transcripts in *Arabidopsis*. To investigate the ability of *SPL15m* to affect expression of its regulatory *miR156* genes, we examined *miR156b* transcript levels in the *sk156* mutant and in complemented *sk156* carrying the *35S:SPL15m* cassette (Table 1). Primers for premature *miR156b* rather than mature *miR156* sequence were used so that measurement of transcripts originating from other *miR156* genes would be excluded. In 30-day-old plants, *miR156b* premature transcript levels were reduced from 82.2-fold above WT levels in *sk156* leaves down to a mean of 7 ± 5.5 fold above WT in the leaves of these complemented lines, with a minimum of 1.4-fold in line 7 and a maximum of 19.6-fold in line 9 (Figure 6G). Thus, *miR156b* transcription was depressed substantially such that ~50% of the complemented lines had WT levels. A reduction of *miR156b* transcript level concurrent with an increase in *SPL15m* transcripts suggests that *SPL15* may have two regulatory functions; one controlling feedback regulation of its cognate regulator *miR156* and one controlling downstream genes, such as *miR172*, *APETALA2-LIKE* (*AP2-like*) transcription factors *TOE1*, and *TOE2*, in the miR156 controlled gene network [9].

### Physical interaction between the SPL15 DNA binding domain and GCAT motifs in the *miR156b* promoter

**Figure 8:**
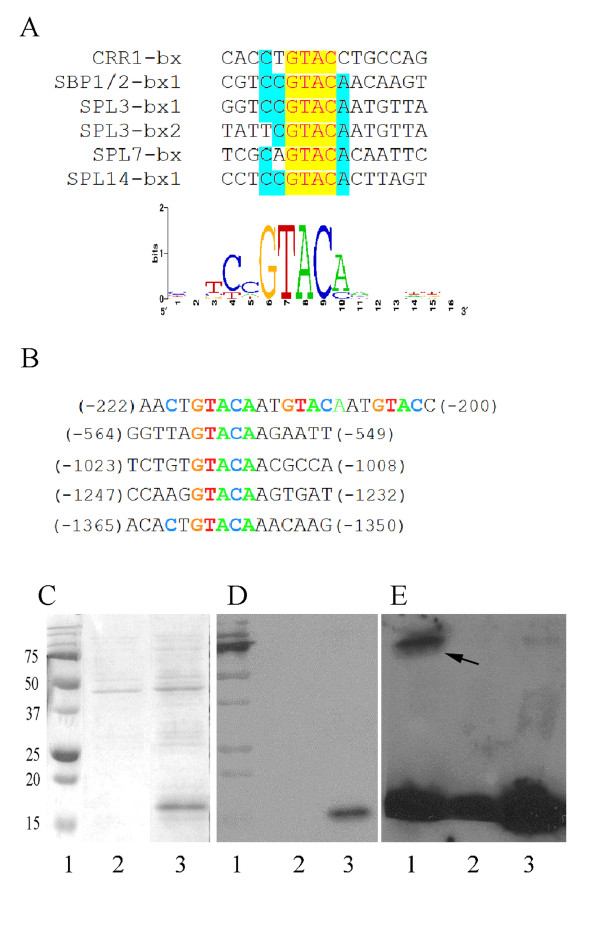
**Interaction of the *miR156 *SBP-binding motif (GTAC core sequence) with the SBP protein domain of *SPL15.*** (**A**) Consensus DNA sequence present in the promoter region of six previously reported genes interacting with SBP DNA binding domains. CCR1-bx represents the GTAC motif binding to SBP protein CCR1 from *Chlamydomonas reinhardtii*[28,29]; SBP1/2-bx1 represents the GTAC motif binding to SBP1 and SBP2 in *Antirrhinum majus*[30]; SPL3-bx1 and -bx2 [5,11], SPL7-bx [32]; and SPL14-bx [31] represents the GTAC motif binding to corresponding SPL proteins from *Arabidopsis thaliana*. Yellow boxes, 100% conserved; green boxes, 67-83% conserved. The degree of conservation is indicated in the schematic by the height of the letters (measured as bits). (**B**) GTAC core repeats present in the *miR156b* promoter sequence (from bp -1 to -1700). (**C)** SDS-PAGE analysis of His-tagged recombinant SBP peptide expressed in *E. coli*. Lane 1, protein marker; lane 2, protein extract of non-induced *E. coli* cells carrying the SBP domain in pET28a; lane 3, protein extract of induced *E. coli* carrying the SBP domain. (**D**) Western blot of resolved proteins in C using anti-His antibody. (**E**) Electrophoretic mobility shift assay illustrating His-tagged SBP peptide bound to a labeled *miR156b* promoter fragment containing tandem repeats of the GTAC core. Lane 1, labeled DNA plus SBP peptide. lane 2, labeled DNA only; lane 3, labeled DNA plus SBP protein and 100-fold excess unlabeled DNA. Black arrow shows the shifted band in lane 1.

To investigate whether SPL15 interacts directly with the *miR156b* promoter to affect transcription, we identified putative SPL15 binding sites in the *miR156b* promoter based on the consensus sequence for SBP binding domain (Figure 8A). To define the consensus sequence, we compared *cis* elements previously identified as SBP box interacting sequences in *Chlamydomonas reinhardtii*[28,29], *Antirrhinum majus*[30] and *A. thaliana*[5,11,31,32] using WebLogo [33]. The consensus SBP domain binding sequence was determined to be 5’-NNGTACR-3’, where frequently N = C and R = A (Figure 8A). This consensus sequence was used to search for putative SPL binding sites in the promoters of *miR156* genes. Putative SPL binding sites with a GTAC core sequence were repeatedly present in the *miR156b* promoter [-1 to -1500], according to the gene transcript start site revealed by Schwab [34]. Of these, a region containing three repeats of the core sequence appeared between -200 bp to -220 bp (Figure 8B) suggesting that *miR156b* expression could be directly controlled by SPL15. This was tested using a His-tagged SPL15 recombinant SBP DNA-binding domain (Figure 8C and D) in an electrophoretic mobility shift assay with a labeled DNA fragment (46-mer) containing a motif from -200 bp to -220 bp from the *miR156b* promoter. The recombinant SBP domain of SPL15*n* specifically bound to this region of the *miR156b* promoter to yield a less mobile promoter fragment (Figure 8E), and competition with 100-fold unlabeled *miR156b* promoter DNA was required to displace the SBP domain. This result suggested that a direct physical interaction can occur between the SPL15 DNA binding domain and the *miR156b* promoter through the GTAC motif.

## Discussion

### Enhanced expression of miR156b in *sk156*

In this study, we highlight a new *Arabidopsis* mutant, *sk156*, with enhanced expression of *miR156b* due to the insertion of a T-DNA activation tag. The elevated *miR156b* transcript levels in *sk156* are responsible for the full spectrum of morphological phenotypes observed for this mutant. Increased seed carotenoid levels and enhanced branching are consistent with phenotypes observed in *B. napus* expressing *AtmiR156b* under the control of the CaMV35S promoter [22]. These results confirm the role for *miR156b* in seed carotenoid accumulation, and are in agreement with morphological changes reported in earlier studies [3,6,12,14,35].

Differences in transcriptional regulation for the native *miR156* promoter, the CaMV35S promoter, and the enhanced *miR156b* native promoter, in addition to different T-DNA insertion sites, can lead to substantial differences in transcript levels between individual transgenic lines and, consequently, to variation in phenotype. For example, *miR156b* transcription driven by the CaVM 35S promoter was more than 2000-fold higher than native *miR156b* transcription in WT plants, whereas *miR156b* transcription in the *sk156* mutant was ~80-fold higher than in WT plants. Such differences in *miR156b* expression levels may result in distinct phenotypes between the *sk156* mutant and *35S:miR156b* lines. For example, numerous small rosettes and tiny leaves were produced in *sk156* (Figure 1C), and comparatively much smaller and many more rosette leaves were noted in the *35S:miR156b* lines in this and previous studies [6].

### Strigolactone-independent seed carotenoid increase in *sk156*

Our data indicated that morphological traits and seed carotenoid profiles are affected in *sk156*. A link between altered carotenoid profiles and branching was previously observed in *max* mutants [17,19] and in an *Arabidopsis* histone methyltransferase (SDG8)-defective mutant [36]. This was largely due to the effect on the biosynthesis of carotenoid-derived strigolactone branching inhibitors [20,21]. In the current study, we also demonstrated that the elevated levels of seed carotenoids in *Arabidopsis* mutant *sk156* were due to the enhancer-driven expression of *miR156b*. However, morphological phenotypes of the T_1_ progeny of *sk156* crossed with WT (in which *CCD7* and *CCD8* are transcribed normally) and of transgenic WT overexpressing *CCD7* or *CCD8* (data not shown) remained almost the same as in *sk156.* Exogenous application of the artificial strigolactone GR24 (2'-epi-5-deoxystrigol) to *sk156* seedlings for six weeks by supplementing the chemical to the *in vitro* plant growing in MS media did not affect *sk156* phenotypes (data not shown). These data suggest that ectopic expression of the *miR156b*-induced branching phenotype in *sk156* might not be directly related to the strigolactone pathway. *In silico* analysis revealed that no known carotenoid biosynthesis or catabolism genes in *Arabidopsis* possess sequences complimentary to the mature sequence of *miR156b*[2]. As well, transcript levels of β-ring carotenoid hydroxylase, ε-ring carotene hydroxylase, lycopene β-cyclase, lycopene ε-cyclase, phytoene desaturase, phytoene synthase or ζ-carotene desaturase were not significantly different in leaves and siliques of *sk156* and WT (data not shown). Thus, miR156b likely affects the seed carotenoid pathway indirectly through the regulation of SPL networks as demonstrated in the miR156-regulated accumulation of anthocyanin via several *SPL* genes [10]. However, we cannot exclude the possibility of changes to seed carotenoid enzyme activities in the absence of transcription changes, such as occured with phytoene synthase in etiolated *Arabidopsis*[37]. Also, the impact on seed carotenoid accumulation may not necessarily be due to biosynthesis, but rather to improved carotenoid sequestration and storage as demonstrated in the *Or* mutant of *Brassica oleracea*[38]. These possibilities point to the need for additional investigation to unravel the biological basis for increased carotenoid accumulation in *sk156* seeds.

In *sk156*, a substantial increase in seed carotenoid levels was detected in all except cryptoxanthin. This minor carotenoid, however, was reduced in *sk156* seeds. Cryptoxanthin may be more efficiently converted to zeaxanthin in *sk156* than in WT *Arabidopsis*. This possibility could arise potentially due to differences in transcription of the related genes between WT and *sk156* and should be tested in the future. For example, in *Zea mays*, a β-carotene hydroxylase variant, ZmBCH1, converts β-carotene to β-cryptoxanthin and zeaxanthin, whereas ZmBCH2 can only convert β-carotene to β-cryptoxanthin [39]. Also, in *Arabidopsis* two variants of β-carotene hydroxylase were found to function differently [40].

A slight decrease in carotenoid abundance was observed in leaves of the *sk156* line even though carotenoid levels were increased in *sk156* seeds. Transgenic *B. napus* in which the gene encoding lycopene ε-cyclase was constitutively down-regulated also showed increased carotenoids in seeds, but not in leaves [41]. Different mechanisms control carotenoid metabolism in green and non-green tissues, including seeds [42-44]. In developing chloroplasts, the synthesis of carotenoids, such as lutein, β-carotene, zeaxanthin and violaxanthin, is regulated in concert with the light-regulated assembly of light-receiving antennae and photosystem centers into which the carotenoids are integrated [45,46]. In contrast, carotenoid compounds in non-green tissue plastids vary widely in quantity and composition and their synthesis is not necessarily regulated similarly to chloroplasts. In seeds, carotenoids are stored in elaioplasts (lipid storing plastids) or amyloplasts (starch storing plastids) [42,47]. Evaluating the impact of *miR156b* on the transcription of genes specifying differentiation into these carotenoid-rich plastid types could lead us to understand why *miR156b* regulates carotenoid accumulation differently in seeds and leaves of *sk*156.

### SPL15 regulation of the morphology and carotenoid alterations in *sk156*

Our 5’ RACE assays showed that *SPL15* mRNA was cleaved at three cleavage sites in the segment located near the middle of the coding sequence and is complementary to mature miR156, suggesting a role for miR156-directed cleavage in *SPL15* transcript processing. However, distinct morphologies were often difficult to find between many of the *Arabidopsis* mutants defective in individual *SPL* genes both in our study and in others [8]. In the present study, *spl15* mutants exhibited slightly stunted shoot growth and a modest increase in carotenoid levels, which were some (but not the full spectrum) of the phenotypes of *sk156*, while the other *spl* mutants examined in this study did not exhibit any discernible phenotypes. Consistently, phenotypes specified by the *sk156* mutant were largely restored to WT phenotypes in *sk156* complemented with *SPL15m* expressed under the control of the CaVM35S promoter or the *AS1* promoter. These pieces of evidence clearly suggest that *miR156* suppresses *SPL15* in the *sk156* mutant and causes much of its phenotypic iteration.

Other *SPL* genes must contribute somewhat to the *sk156* mutant phenotype development, since *spl15* mutants did not show the entire spectrum of *sk156* phenotypes and *sk156* was not fully restored to a WT phenotype by *SPL15m* expressed from a native *SPL15* promoter. These data not only indicate that SPL15 is functionally redundant with other miR156-targeted *SPL* genes, but that this redundancy probably is limited by the endogenous regulation of temporal and spatial gene expression. Our data derived from *AS1* promoter-driven *SPL15m* experiments show that the leaf primordium within the shoot apex is a crucial site for *SPL15m* expression to restore *sk156* phenotypes. This is consistent with previous findings that the shoot apex is a predominant expression site for some *SPL* genes [2,5,13] and that *AS1* promoter-driven *miR156* promotes leaf initiation by suppressing *SPL* genes at the shoot apical meristem [14]. *AS1* is expressed only in emerging lateral organ primordia, and in seeds its expression is detectable only in limited subepidermal cells corresponding to cotyledon initials at the heart stage [48]. In our hands, *AS1:GUS* + plants did not show visible GUS staining in seeds but did show staining in shoot tips, suggesting that *SPL15m* transcript level might not be significantly elevated in *AS1:SPL15m*^+^ seeds compared to *sk156*. The morphological changes in transgenic *sk156* expressing *35S/AS1:SPL15m* point to functional redundancy of *SPL15* with at least *SPL9* and *SPL10* on plastochron length [8], *SPL9* on shoot development [13], and *SPL3*, *SPL4*, and *SPL5* on flowering stage transition [4,12].

Expression of miR156b-insensitive *SPL15m* modifies leaf shape in the *sk156* lines carrying *35S:SPL15m* or *SPL15p:SPL15m* constructs. A role for SPL15 in leaf morphological traits was also revealed by Usami et al [49]. The enhancement of *miR156b* expression in *sk156* possibly prevents the plants from maintaining sufficient *SPL* transcript levels required for normal control of leaf architecture, including those from *SPL15*. These findings are consistent with reduced expression of 10 *SPL* genes in the inflorescences of *Arabidopsis* lines over-expressing *miR156b*[6]. Expression of *SPL15* gene in leaf primodia was also important for rescuing WT-like seed carotenoid and morphology traits in *sk156*. This was supported partially by the finding that leaf primodia are a site where SPL factors control the rate of leaf initiation [8].

### SPL15 feedback regulation of miR156b

Feedback loops, in which a miRNA-targeted transcription factor either increases or decreases the expression of its cognate miRNA, have been reported in animals [50,51]. However, the mechanism underlying this feedback regulation is unclear, particularly in plants. In our hands, complementation of *sk156* with *SPL15m* leads to increased levels of *SPL15* transcripts and decreased levels of *miR156b* transcripts. The reduction in *miR156b* expression due to increased *SPL15* was consistent with the inverse correlation of *SPL3* and mature *miR156* transcripts in WT *Arabidopsis* at the early vegetative stages (19- and 26-d-old) [12]. These data suggest that negative feedback regulation by SPL15 may exist for *miR156b*. A recent study on the regulation of *miR156a* and *miR172b* in early vegetative stages proposed feedback regulation between the miRNAs and their targets but did not further investigate the underlying mechanism [9].

All SPL family members contain a conserved SBP DNA binding domain [5,30] with variations outside the domain. Moreover, DNA sequences in promoters of SPL-regulated genes contain a GTAC core [5,28,29,31,32,52], which is a key element for transcription regulation [53]. Our gel shift assay demonstrated a direct physical interaction between the SPL15 SBP domain and the promoter region of *miR156b* containing three repeats of the GTAC core sequence. This suggests a mechanism for the underlying feedback regulation of *miR156b* by its target SPL15. Support for this finding comes from reports by Birkenbihl et al [52] and Yamasaki et al [32] which indicated that such an interaction also exists between the SBP domain for SPL1, -3, -7 and -8 and the GTAC element.

## Conclusions

In the present study we showcase a new mutant, *sk156*, in which a T-DNA insert containing four CaMV35S enhancers hyper-induced *miR156b*. We documented phenotypes for several *sk156* lines complemented with different expression cassettes for *miR156b*-sensitive and *miR156b*-insensitive *SPL15,* and showed for the first time a direct interaction between SPL15 and *miR156b*. These data and the inverse pattern of *miR156b* and *SPL15* expression in mutant *sk156* lead us to conclude that negative feedback regulation of *miR156b* by SPL15 exists. A second finding is that *miR156b* regulates seed carotenoid abundance in *Arabidopsis* differently than in leaves although the underlying molecular basis remains a subject for further investigation.

## Methods

### Plants and growth conditions

The *sk156* mutant was isolated from a T-DNA activation-tagged mutant population of *Arabidopsis* Col-4 [23]. The population was generated at the AAFC Saskatoon Research Centre using the pSKI015 binary vector containing a T-DNA with a *Bar* gene and 4 tandem CaMV enhancers [54]. SALK and FLAG T-DNA insertion lines with knockouts in *SPL* genes were obtained from the Arabidopsis Biological Resource Center and the FST (flanking insertion sites) project ( http://www.arabidopsis.org; Additional file 2). *max* mutants were kindly provided by Dr. M-S Peng (University of Guelph) and transgenic *Arabidopsis* lines over-expressing *miR156b* under the control of the CaMV35S promoter were provided by Dr. R. S. Poethig (University of Pennsylvania). To recover homozygous mutant lines, segregating offspring grown were grown on half-strength Murashige-Skoog (MS) basal medium (Sigma, M5519-50L) with 0.8% agar and a selective agent (7.5 mg/L phosphinothricin or 50 mg/L kanamycin) and were screened by PCR using primers specific for each insertion site (Additional file 5). Homozygous plants were transplanted into growth chamber pots for further characterization.

For phenotypic analysis, plants were grown in 32-well flats containing Co-Co Mix soil mixture consisting of compacted coconut fibre/peat moss/vermiculite (1/3/3, v/v*/*v) and 15-9-12 "Osmocote PLUS" controlled release fertilizer (Scotts Company LLC). Plant age was recorded from the time seeds were imbibed. All plants were kept in a controlled environment growth chamber under a 16/8 h light/dark photoperiod with a light intensity of 230 μE/min/m^2^ and temperature of 20^o^C/17^o^C. Leaf and branch number means were calculated from 30 individual plants of mutant or WT lines. A Duncan's multiple range test was conducted to show significant differences between the means at p < 0.01 and p < 0.05.

### Carotenoid extraction and quantification

For carotenoid analysis, leaves of 28-day-old plants were excised and samples immediately ground in liquid nitrogen. Mature seeds harvested from different plants were kept at -80^o^C for further analysis. Tissues were maintained at -80^o^C before extraction to minimize carotenoid degradation. Approximately 100 mg of leaf tissue powder and 150 mg of homogenized seeds were used for carotenoid extraction. Carotenoid extraction and HPLC quantitative analysis were carried out as previously described for seeds [41]. HPLC chromatograph peaks were identified by comparing their retention times and absorption spectra to authentic standards, and quantified using standard curves corresponding to carotenoid compounds. Pure chemical standards for β-carotene and lutein were purchased from Sigma. β-cryptoxanthin, zeaxanthin and violaxanthin were purchased from CaroteNature (Lupsingen, Switzerland). Each sample was pooled from the leaves or seeds of 6-10 different plants. Six replicates (two biological replicates with three technical replicates) were used for carotenoid analysis. A Duncan's multiple range test was used to separate statistically different means at p < 0.01 and p < 0.05.

### Molecular characterization of the *sk156* mutant

Plant genomic DNA was extracted to determine T-DNA copy number in the *sk156* mutant. Southern analysis was conducted using 15 μg of genomic DNA digested with *Bam*HI and *Hind*III. The 1.4 kb fragment was excised from a pSKI015 plasmid using *Hind*III and *EcoR*I, labeled with ^32^P, and used as a hybridization probe. To identify the T-DNA insertion site of *sk156*, genomic DNA was digested with each of four blunt-end restriction enzymes provided in the GenomeWalk kit (Clontech), ligated with adaptors, and flanking regions amplified by nested PCR with the T-DNA specific primers pSKI015-GW-LB1 and pSKI015-GW-LB2 according to the manufacturer’s instructions (Additional file 5). Cloned GenomeWalk PCR products were sequenced using the pSKI015-GW-LB2 primer (Additional file 5) and the T-DNA insertion site was determined by BLAST analysis to the TAIR sequence database (Version 9) ( http://www.arabidopsis.org/Blast/index.jsp). Genetic segregation of plants with T-DNA inserts was completed in triplicate by recording T-DNA insertion and the number of surviving seedlings that geminated out of ~100 seeds growing in half-strength MS media containing 7.5 mg/L phosphinotricin or 50 mg/L kanamycin, followed by χ^2^ analysis.

### Constructs and plant transformation

Total RNA for cDNA synthesis was extracted from 3-week-old *Arabidopsis thaliana* ecotype Columbia plants using the QIAGEN RNeasy kit (Qiagen) according to the manufacturer’s instructions. DNase I was used for on-column DNA digestion to minimize genomic DNA contamination. First-strand cDNA was synthesized by reverse transcription of 300 ng of total RNA in a final reaction volume of 20 μL using random primers and 200 units of SuperScript™ II Reverse Transcriptase (Invitrogen). Full length cDNA of *miR156b*, *SPL15,* AT4G30975 and *bHLH069* was amplified using Platinum® Taq High Fidelity DNA Polymerase (Invitrogen) and gene specific primer pairs (miR156b_XbaI_F/ miR156b_SacI_R; pXbaI_ SPL15F/ pSacI _SPL15R; p30975BamHI_F /p30975SacI_R; and pbHLH_F / pbHLH_R, respectively), with addition of restriction enzyme sites at the 5’ and 3’ ends of the genes (Additional file 5). *SPL15m* was generated by introducing 11 mutations into the predicted *miR156* binding site using the PCR primers pSPL15m851F and pSPL15m865R containing mutated sequences (Additional file 5). The *AS1* promoter was isolated from *A. thaliana* Columbia genomic DNA using pAS1_HindIII_F and pAS1_XbaI_R (Additional file 5) according to Wang et al [8], while a 3 kb DNA fragment upstream of the *SPL15* transcription start site including both the ‘proximal promoter’ and ‘distal promoter’ regions [55] was amplified by PCR using the primers pSPL15Pr3_HindIII_F and pSPL15Pr3_XbaI_R (Additional file 5) and used as the native *SPL15* promoter. Amplified PCR fragments were cloned into pCR2.1-TOPO vector (Invitrogen) and verified by DNA sequencing. The fragments were retrieved with corresponding restriction enzymes and inserted into the binary vector pBI121 digested with the same pair of restriction enzymes (replacing the original *gusA* gene or *CaMV35S* promoter in the vector). The resulting binary expression vectors contained an *nptII* gene and transgenes under the control of either the *CaMV35S* promoter, the *AS1* promoter, or the native *SPL15* promoter and were introduced into *Agrobacterium tumefaciens* GV3101pMP90 and used to transform *Arabidopsis* using the standard floral dip method. Putative transgenic lines that survived antibiotic selection were transplanted into the greenhouse for further analysis.

### 5’RACE

5’RACE was carried out using the FirstChoice RLM RACE Kit (Ambion) following the manufacturer's instructions specially for the detection of miRNA degradation products [56]. Briefly, total RNA was isolated from 3-week-old seedlings as described above. Five micrograms of total RNA was ligated to the RNA adapter and a random-primed reverse transcription reaction was performed to synthesize cDNA. A second round of PCR was carried out using a nested adapter primer and primers specific for *SPL15* (Additional file 5). The RACE products were cloned into the pGEM T Easy vector (Promega) for sequencing.

### Quantitative Real Time PCR analysis

Total RNA extraction, on-column genomic DNA digestion, and first-strand cDNA synthesis were conducted as described above. Real time quantitative RT-PCR (qPCR) analyses were performed with gene specific primers listed in supplemental Table S1. To quantify miR156b, primers based on the stem sequence of the pre-miR156b hairpin structure were designed to measure premature miR156b according to Schmittgen et al. [57] (Additional file 5). qPCR mixtures contained 10 μl of diluted cDNA, 12.5 μl of 2X SYBR Green qPCR Master Mix (Cat No. 11735-040, Invitrogen) and 200 nM of each gene-specific primer in a final volume of 25 μl. qPCR reactions were conducted using the StepOne Plus system and software (Applied Biosystems) using a relative standard curve method and default reaction parameters. The relative index of the standard curve was over 98%. Control PCR reactions with no templates were also performed for each primer pair. The specificity of amplicons was verified by melting curve analysis (60 to 95^o^C) after 40 cycles and by agarose gel electrophoresis. All samples were assayed in triplicate from two independent RNA preparations. Mean expression values of all replicates were calculated and normalized to the expression of *PEROXIN 4* (*PEX4*, set at 1), a suitable endogenous reference gene because of its stable and low level expression in *Arabidopsis*[58]. All PCR reactions displayed efficiencies between 87 and 115%. Normalized means were analyzed for significant differences by a Duncan’s test (p < 0.05).

### Expression and extraction of SBP-domains

For recombinant protein expression in *Escherichia coli*, a coding sequence fragment encoding the SPL15 SBP domain (80 amino acid residues) plus 5 amino acid residues both upstream and downstream [48] was amplified by PCR using the primers p15SBP-BamHI-F and p15SBP-SalI-R with addition of *Bam*HI at the 5’ end and *Sal*I and a TGA stop codon at the 3’ end (Additional file 5). The PCR product was digested with *Bam*HI and *Sal*I and cloned into the pET28a vector (Novagen) between the *Bam*HI and *Sal*I sites. The resulting chimeric recombinant protein fused with a His tag (16.3kD) was expressed in *E. coli* strain Rosetta 2(DE3) pLacI (Clontech) by induction with 0.8 mM isopropyl-β-d-thiogalactopyranoside. Cells were harvested by centrifugation and resuspended in BugBuster® Master Mix (Novagen) to lyse the cells. Recombinant His-tagged SBP proteins were detected in the inclusion bodies using anti-His antibody (G-18) (Santa Cruz Biotechnology) in Western blot assays. Proteins in inclusion bodies were solubilized with 1.5% sarkosyl (N-laurylsarcosine) according to Frangioni and Neel [59], and recombinant SBP protein recovered and re-folded using a protein refolding kit (TB234, Novagen) according to the manufacturer’s instructions. Protein concentration was measured in lysates by a NanoDrop 1000 spectrophotometer (Thermo Fisher Scientific) and proteins separated in 13% SDS–polyacrylamide gels. The protein was stored at 4°C until use in gel shift assays.

### Electrophoretic mobility shift assays

The ability of SPL15 SBP to bind to the *miR156b* promoter DNA was examined using electrophoretic mobility shift assays. A 46 residue DNA fragment, which included the three repeated GTAC core sequences close to the transcriptional start site of *miR156b* (shown in Figure 8B), was synthesized using primers R156b-bx1-U and R156b-bx1-L (Additional file 5), hybridized and labeled with digoxigenin using a DIG Gel Shift kit (second generation) (Roche). The labeled DNA fragment (~20 fmol) was incubated for 30 min at 25°C with or without the SPL15 SBP protein (~500 ng) in 20 μL of reaction buffer containing 10 mM Tris (pH 7.5), 50 mM KCl, 5 mM MgCl, 5 mM DTT, 2.5% glycerol, and 0.05% NP-40. Then, a 100-fold excess of the unlabeled promoter DNA fragment was added to the reactions. After incubation, the mixtures were separated by polyacrylamide electrophoresis (7.5% gel) at 4^o^C for 1.5 h (0.8 V/cm^2^) in 0.5 X TBE (44.5 mM Tris base, 44.5 mM boric acid, and 1 mM EDTA at pH 8.0). DNA was blotted onto a nylon membrane and mobility changes for the labeled *miR156b* promoter fragment detected with digoxigenin-specific antibodies.

## Authors' contributions

SW designed the experiments and conducted the majority of the experimentation, analyzed data, and drafted the manuscript; IAP Parkin constructed and provided the *Arabidopsis* activation-tagged population; BY was involved in construction of SPL15 binary vectors; MJG performed computational sequence analysis of the miR156b promoter binding motif; MYG, and AH provided critical feedback on experimental concepts. MYG, GGK, DDH and AH revised the manuscript. All authors read and approved the final manuscript.

## Authors’ information

Submitting author: Shu Wei, PhD in Plant Sciences and currently Professor in Plant Molecular Biology and Biotechnology at Anhui Agricultural University, China, working on, but not limited to plant microRNA regulated gene networks and metabolic pathway engineering.

## Supplementary Material

Additional file 1 **Schematic diagram of disrupted carotenoid cleavage dioxygenase genes *CCD7 *and *CCD8 *in the *max3-9 *and *max4-1 *mutants used in this study.** Boxes represent exons and lines represent introns. Triangles show the T-DNA insertion sites. The *max* mutants were previously reported by (Booker et al. 2004). Click here for file

Additional file 2 **SALK and FLAG T-DNA insertion lines for miR156-targeted *SPL *genes.** Click here for file

Additional file 3 **Reduced lengths of cauline stem basal internodes in three *spl15 *mutants compared with WT *Arabidopsis*.** Length of the cauline stem basal inter-node was measured from the rosette core up to the first visible basal node for WT, three *spl* mutants and *sk156* plants grown for 6 weeks. Click here for file

Additional file 4 **Confirmation by PCR of transgene presence in *sk156 *lines transformed with a *35S:SPL15m *gene.** The miR156 insensitive *SPL15m* contained 11 mutated nucleotides as described in Materials and Methods. Primer sequences are listed in Additional file 5. (A) Activation-tag from pSKI015 T-DNA detected in transgenic *sk156* plants carrying *35S:SPL15m* cassette (lanes 0-11) and in the *sk156* background alone (lanes 12 and 13). Primers SK2222-F (430bp upstream) and SK2222-R (1830bp downstream) flanking the T-DNA insertion site and primer pSKI015-GW-LB2 (439bp to the T-DNA left border) were used to detect the insert. In WT lane, no T-DNA insert was detected and only a fragment close to 1.9kb was present due to genomic DNA amplified with the primers flanking the T-DNA insertion site. In homozygous *sk156* plants which did not carry *35S:SPL15m,* a single T-DNA fragment (869bp) was generated. 1kb, 1-kb Plus DNA ladder (Invitrogen); WT, Col-4; pSKI015, plasmid containing the activation tag present in *sk156*. (B) Transgene *SPL15m* detected in the transgenic *sk156* lines carrying *35S:SPL15m* (lanes 0-14), but not in sk156 alone using primers 35SF3 and SPL15R. Click here for file

Additional file 5 Primers used in this study. Click here for file

Additional file 6 **PCR confirmation of transgene presence in four different miR156-sensitive or miR156-insensitive transgenic *Arabidopsis *populations used in this manuscript.** (**A**) Lanes 2-8, PCR product (818bp) for 7 transgenic plants carrying a *35S:AT4G30975* cassette in a WT background using primers 35S-F3 and p795-3R. P, binary plasmid pBI121 containing *35S:AT4G30975* as a positive control. WT, Col-4. (**B**), (**C**) and (**D**) Transgene PCR product (636bp) carrying *AS1:SPL15m*, *AS1:SPL15n* and *SPL:SPL15m* cassettes in a *sk156* background, respectively, using primers SPL15-871F and NosTer-R6. P, plasmid containing *35S:SPL15* as a positive control. Black arrows points to the DNA marker. Primer sequences are listed in Additional file 5. 1kb, 1-kb Plus DNA ladder (Invitrogen). Click here for file

## References

[B1] Zhang B Pan X Cannon CH Cobb GP Anderson TA Conservation and divergence of plant microRNA genes Plant J 2006 46 243 259 10.1111/j.1365-313X.2006.02697.x 16623887

[B2] Rhoades MW Reinhart BJ Lim LP Burge CB Bartel B Bartel DP Prediction of plant microRNA targets Cell 2002 110 513 520 10.1016/S0092-8674(02)00863-2 12202040

[B3] Xie K Wu C Xiong L Genomic organization, differential expression, and interaction of SQUAMOSA promoter-binding-like transcription factors and microRNA156 in rice Plant Physiol 2006 142 280 293 10.1104/pp.106.084475 16861571 PMC1557610

[B4] Gandikota M Birkenbihl RP Hohmann S Cardon GH Saedler H Huijser P The miRNA156/157 recognition element in the 3’UTR of the Arabidopsis SBP box geneSPL3prevents early flowering by translational inhibition in seedlings Plant J 2007 49 683 693 10.1111/j.1365-313X.2006.02983.x 17217458

[B5] Cardon G Höhmann S Klein J Nettesheim K Sardler H Huijser P Molecular characterisation of theArabidopsisSBP-box genes Gene 1999 237 91 104 10.1016/S0378-1119(99)00308-X 10524240

[B6] Schwab R Palatnik JF Riester M Schommer C Schmid M Weigel D Specific effects of microRNAs on the plant transcriptome Dev Cell 2005 8 517 527 10.1016/j.devcel.2005.01.018 15809034

[B7] Guo AY Zhu QH Gu X Ge S Yang J Luo J Genome-wide identification and evolutionary analysis of the plant specific SBP-box transcription factor family Gene 2008 418 1 8 10.1016/j.gene.2008.03.016 18495384

[B8] Wang JW Czech B Weigel D miR156-Regulated SPL transcription factors define an endogenous flowering pathway inArabidopsis thaliana Cell 2009 138 738 749 10.1016/j.cell.2009.06.014 19703399

[B9] Wu G Park MY Conway SR Wang J-W Weigel D Poethig RS The sequential action of miR156 and miR172 regulates developmental timing inArabidopsis Cell 2009 138 750 759 10.1016/j.cell.2009.06.031 19703400 PMC2732587

[B10] Gou J-Y Felippes FF Liu C-J Weigel D Wang J-W Negative regulation of anthocyanin biosynthesis inArabidopsisby a miR156-targeted SPL transcription factor Plant Cell 2011 23 1512 1522 10.1105/tpc.111.084525 21487097 PMC3101539

[B11] Cardon G Höhmann S Nettesheim K Saedler H Huijser P Functional analysis of theArabidopsis thalianaSBP-box geneSPL3: A novel gene involved in the floral transition Plant J 1997 12 367 377 10.1046/j.1365-313X.1997.12020367.x 9301089

[B12] Wu G Poethig RS Temporal regulation of shoot development inArabidopsis thalianabymiR156and its targetSPL3 Development 2006 133 3539 3547 10.1242/dev.02521 16914499 PMC1610107

[B13] Schwarz S Grande AV Bujdoso N Saedler H Huijser P The microRNA regulated SBP-box genesSPL9andSPL15control shoot maturation in Arabidopsis Plant Mol Biol 2008 67 183 195 10.1007/s11103-008-9310-z 18278578 PMC2295252

[B14] Wang JW Schwab R Czech B Mica E Weigel D Dual effects of miR156-targetedSPLgenes andCYP78A5/KLUHon plastochron length and organ size inArabidopsis thaliana Plant Cell 2008 20 1231 1243 10.1105/tpc.108.058180 18492871 PMC2438454

[B15] Shikata M Koyama T Mitsuda N Ohme-Takagi M Arabidopsis SBP-box genesSPL10,SPL11andSPL2control morphological change in association with shoot maturation in the reproductive phase Plant Cell Physiol 2009 50 2133 2145 10.1093/pcp/pcp148 19880401

[B16] Yu N Cai WJ Wang S Shan CM Wang LJ Chen XY Temporal control of trichome distribution by microRNA156-targetedSPLgenes inArabidopsis thaliana Plant Cell 2010 22 2322 2335 10.1105/tpc.109.072579 20622149 PMC2929091

[B17] Booker J Auldridge M Wills S McCarty D Klee H Leyser O MAX3/CCD7 is a carotenoid cleavage dioxygenase required for the synthesis of a novel plant signaling molecule Curr Biol 2004 14 1232 1238 10.1016/j.cub.2004.06.061 15268852

[B18] Booker J Sieberer T Wright W Williamson L Willett B Stirnberg S Turnbull C Srinivasan P Goddard P Leyser O MAX1encodes a cytochrome P450 family member that acts downstream ofMAX3/4to produce a carotenoid-derived branch-inhibiting hormone Del Cell 2005 8 443 449 10.1016/j.devcel.2005.01.009 15737939

[B19] Sorefan K Booker J Haurogné K Goussot M Bainbridge K Foo E Chatfield S Ward S Beveridge C Rameau C Leyser O MAX4andRMS1are orthologous dioxygenase-like genes that regulate shoot branching inArabidopsisand pea Genes Dev 2003 17 1469 1474 10.1101/gad.256603 12815068 PMC196077

[B20] Umehara M Hanada A Yoshida S Akiyama K Arite T Takeda-Kamiya N Magome H Kamiya Y Shirasu K Yoneyama K Kyozuka J Yamaguchi S Inhibition of shoot branching by new terpenoid plant hormones Nature 2008 455 195 200 10.1038/nature07272 18690207

[B21] Gomez-Roldan V Fermas S Brewer PB Puech-Pagès V Dun EA Pillot JP Letisse F Matusova R Danoun S Portais JC Bouwmeester H Bécard G Beveridge CA Rameau C Rochange SF Strigolactone inhibition of shoot branching Nature 2008 455 189 194 10.1038/nature07271 18690209

[B22] Wei S Yu B Gruber MY Khachatourians GG Hegedus DD Hannoufa A Enhanced seed carotenoid levels and branching in transgenicBrassica napusexpressing theArabidopsis miR156bgene J Agric Food Chem 2010 58 9572 9578 10.1021/jf102635f 20707346

[B23] Robinson SJ Tang LH Mooney BA McKay SJ Clarke WE Links MG Karcz S Regan S Wu YY Gruber MY Cui D Yu M Parkin IA An archived activation tagged population ofArabidopsis thalianato facilitate forward genetics approaches BMC Plant Biol 2009 9 101 10.1186/1471-2229-9-101 19646253 PMC3091532

[B24] Auldridge ME Block A Vogel JT Dabney-Smith C Mila I Bouzayen M Magallanes-Lundback M DellaPenna D McCarty DR Klee HJ Characterization of three members of the Arabidopsis carotenoid cleavage dioxygenase family demonstrates the divergent roles of this multifunctional enzyme family Plant J 2006 45 982 993 10.1111/j.1365-313X.2006.02666.x 16507088

[B25] Heim MA Jakoby M Werber M Martin C Weisshaar B Bailey PC The basic helix-loop-helix transcription factor family in plants: a genome-wide study of protein structure and functional diversity Mol Biol Evol 2003 20 735 747 10.1093/molbev/msg088 12679534

[B26] Riechmann JL Heard J Martin G Reuber L Jiang C Keddie J Adam L Pineda O Ratcliffe OJ Samaha RR Creelman R Pilgrim M Broun P Zhang JZ Ghandehari D Sherman BK Yu G Arabidopsistranscription factors: genome-wide comparative analysis among eukaryotes Science 2000 290 2105 2110 10.1126/science.290.5499.2105 11118137

[B27] Eshed Y Baum SF Perea JV Bowman JL Establishment of polarity in lateral organs of plants Curr Biol 2001 11 1251 1260 10.1016/S0960-9822(01)00392-X 11525739

[B28] Quinn JM Barraco P Eriksson M Merchant S Coordinate copper- and oxygen-responsiveCyc6andCpx1expression inChlamydomonasis mediated by the same element J Biol Chem 2000 275 6080 6089 10.1074/jbc.275.9.6080 10692397

[B29] Kropat J Tottey S Birkenbihl RP Depège N Huijser P Merchant S A regulator of nutritional copper signaling inChlamydomonasis an SBP domain protein that recognizes the GTAC core of copper response element Proc Natl Acad Sci U S A 2005 102 18730 18735 10.1073/pnas.0507693102 16352720 PMC1311908

[B30] Klein J Saedler H Huijser P A new family of DNA binding proteins includes putative transcriptional regulators of theAntirrhinum majusfloral meristem identity geneSQUAMOSA Mol Gen Genet 1996 250 7 16 8569690 10.1007/BF02191820

[B31] Liang X Nazarenus TJ Stone JM Identification of a consensus DNA-binding site for theArabidopsis thalianaSBP domain transcription factor, AtSPL14, and binding kinetics by surface plasmon resonance Biochemistry 2008 47 3645 3653 10.1021/bi701431y 18302343

[B32] Yamasaki H Hayashi M Fukazawa M Kobayashi Y Shikanai T SQUAMOSApromoter binding protein-like7 is a central regulator for copper homeostasis inArabidopsis Plant Cell 2009 21 347 361 10.1105/tpc.108.060137 19122104 PMC2648088

[B33] Crooks G Hon G Chandonia JM Brenner SE WebLogo: a sequence logo generator Genome Res 2004 14 1188 1190 10.1101/gr.849004 15173120 PMC419797

[B34] Schwab R Functions and target selection of Arabidopsis microRNAs 2006 Tübingen Germany: Eberhard Karls University PhD thesis

[B35] Chuck G Cigan AM Saeteurn K Hake S The heterochronic maize mutantCorngrass1results from overexpression of a tandem microRNA Nat Genet 2007 39 544 549 10.1038/ng2001 17369828

[B36] Cazzonelli CI Cuttriss AJ Cossetto SB Pye W Crisp P Whelan J Finnegan EJ Turnbull C Pogson BJ Regulation of carotenoid composition and shoot branching inArabidopsisby a chromatin modifying histone methyltransferase, SDG8 Plant Cell 2009 21 39 53 10.1105/tpc.108.063131 19174535 PMC2648095

[B37] Rodríguez-Villalón A Gas E Rodríguez-Concepción M Phytoene synthase activity controls the biosynthesis of carotenoids and the supply of their metabolic precursors in dark-grown Arabidopsis seedlings Plant J 2009 60 424 435 10.1111/j.1365-313X.2009.03966.x 19594711

[B38] Paolillo DJ Jr Garvin DF Parthasarathy MV The chromoplasts of Or mutants of cauliflower (Brassica oleracea. Var. Botrytis) Protoplasma 2004 224 245 253 10.1007/s00709-004-0059-1 15614485

[B39] Li Q Farre G Naqvi S Breitenbach J Sanahuja G Bai C Sandmann G Capell T Christou P Zhu C Cloning and functional characterization of the maize carotenoid isomerase and β-carotene hydroxylase genes and their regulation during endosperm maturation Transgenic Res 2010 19 1053 1068 10.1007/s11248-010-9381-x 20221689

[B40] Tian L DellaPenna D Characterization of a second carotenoid βhydroxylase gene from Arabidopsis and its relationship to theLUT1locus Plant Mol Biol 2001 47 379 388 10.1023/A:1011623907959 11587509

[B41] Yu B Lydiate DJ Schäfer UA Hannoufa A Characterization of a β-carotene hydroxylase ofAdonis aestivalisand its expression inArabidopsis thaliana Planta 2007 226 181 192 10.1007/s00425-006-0455-1 17171373

[B42] Howitt CA Pogson BJ Carotenoid accumulation and function in seed and non-green tissues Plant Cell Environ 2006 29 435 445 10.1111/j.1365-3040.2005.01492.x 17080597

[B43] Galpaz N Ronen G Khalfa Z Zamir D Hirschberg J A chromoplast-specific carotenoid biosynthesis revealed by cloning of the tomatowhite-flowerlocus Plant Cell 2006 18 1947 1960 10.1105/tpc.105.039966 16816137 PMC1533990

[B44] Lopez AB Van Eck J Conlin BJ Paolillo DJ O'Neill J Li L Effect of the cauliflowerOrtransgene on carotenoid accumulation and chromoplast formation in transgenic potato tubers J Exp Bot 2008 59 213 223 10.1093/jxb/erm299 18256051

[B45] Young AJ Young AJ, Britton G Factors that affect the carotenoid composition of higher plants and algae Carotenoids in photosynthesis 1993 London: Chapman and Hall 161 205

[B46] Römer S Lübeck J Kauder F Steiger S Adomat C Sandmann G Genetic engineering of a zeaxanthin-rich potato by antisense inactivation and co-suppression of carotenoid epoxidation Metab Eng 2002 4 263 272 10.1006/mben.2002.0234 12646321

[B47] Vishnevetsky M Ovadis M Vainstein A Carotenoid sequestration in plants: the role of carotenoid-associated proteins Trends Plant Sci 1999 4 232 235 10.1016/S1360-1385(99)01414-4 10366880

[B48] Byrne ME Barley R Curtis M Arroyo JM Dunham M Hudson A Martienssen RA Asymmetric leaves1mediates leaf patterning and stem cell function inArabidopsis Nature 2000 408 967 971 10.1038/35050091 11140682

[B49] Usami T Horiguchi G Yano S Tsukaya H Themore and smaller cellsmutants ofArabidopsis thalianaidentify novel roles forSQUAMOSA PROMOTER BINDING PROTEIN-LIKEgenes in the control of heteroblasty Development 2009 136 955 964 10.1242/dev.028613 19211679

[B50] Kim J Inoue K Ishii J Vanti WB Voronov SV Murchison E Hannon G Abeliovich A A MicroRNA feedback circuit in midbrain dopamine neurons Science 2007 317 1220 1224 10.1126/science.1140481 17761882 PMC2782470

[B51] Varghese J Cohen SM microRNA miR-14 acts to modulate a positive autoregulatory loop controlling steroid hormone signaling inDrosophila Genes Dev 2007 21 2277 2282 10.1101/gad.439807 17761811 PMC1973141

[B52] Birkenbihl RP Jach G Saedler H Huijser P Functional dissection of the plant-specific SBP-domain: overlap of the DNA-binding and nuclear localization domains J Mol Biol 2005 352 585 596 10.1016/j.jmb.2005.07.013 16095614

[B53] Moseley JL Page MD Alder NP Eriksson M Quinn J Soto F Theg M Hippler M Merchant S Reciprocal expression of two candidate di-iron enzymes affecting photosystem I and light-harvesting complex accumulation Plant Cell 2002 14 673 688 10.1105/tpc.010420 11910013 PMC150588

[B54] Weigel D Ahn JH Blázquez MA Borevitz JO Christensen SK Fankhauser C Ferrándiz C Kardailsky I Malancharuvil EJ Neff MM Nguyen JT Sato S Wang ZY Xia Y Dixon RA Harrison MJ Lamb CJ Yanofsky MF Chory J Activation tagging in Arabidopsis Plant Physiol 2000 122 1003 1014 10.1104/pp.122.4.1003 10759496 PMC1539247

[B55] Shahmuradov IA Gammerman AJ Hancock JM Bramley PM Solovyev VV PlantProm: a database of plant promoter sequences Nucleic Acids Res 2003 31 114 117 10.1093/nar/gkg041 12519961 PMC165488

[B56] Wang XJ Reyes JL Chua NH Gaasterland T Prediction and identification ofArabidopsis thalianamicroRNAs and their mRNA targets Genome Biol 2004 5 R65 10.1186/gb-2004-5-9-r65 15345049 PMC522872

[B57] Schmittgen TD Jiang J Liu Q Yang L A high-throughput method to monitor the expression of microRNA precursors Nucleic Acids Res 2004 32 e43 10.1093/nar/gnh040 14985473 PMC390315

[B58] Czechowski T Stitt M Altmann T Udvardi MK Scheible WR Genome-wide identification and testing of superior reference genes for transcript normalization in Arabidopsis Plant Physiol 2005 139 5 17 10.1104/pp.105.063743 16166256 PMC1203353

[B59] Frangioni JV Neel BG (1993) Solubilization and purification of enzymatically active glutathione S-transferase (pGEX) fusion proteins Anal Biochem 1993 210 179 187 10.1006/abio.1993.1170 8489015

