# Supplementary figures and images for: Arabidopsis mutant sk156 reveals complex regulation of SPL15 in a miR156-controlled gene network
Source: BMC Plant Biol. 2012 Sep 18;12:169. doi: 10.1186/1471-2229-12-169 (PMC3520712; doi:10.1186/1471-2229-12-169)

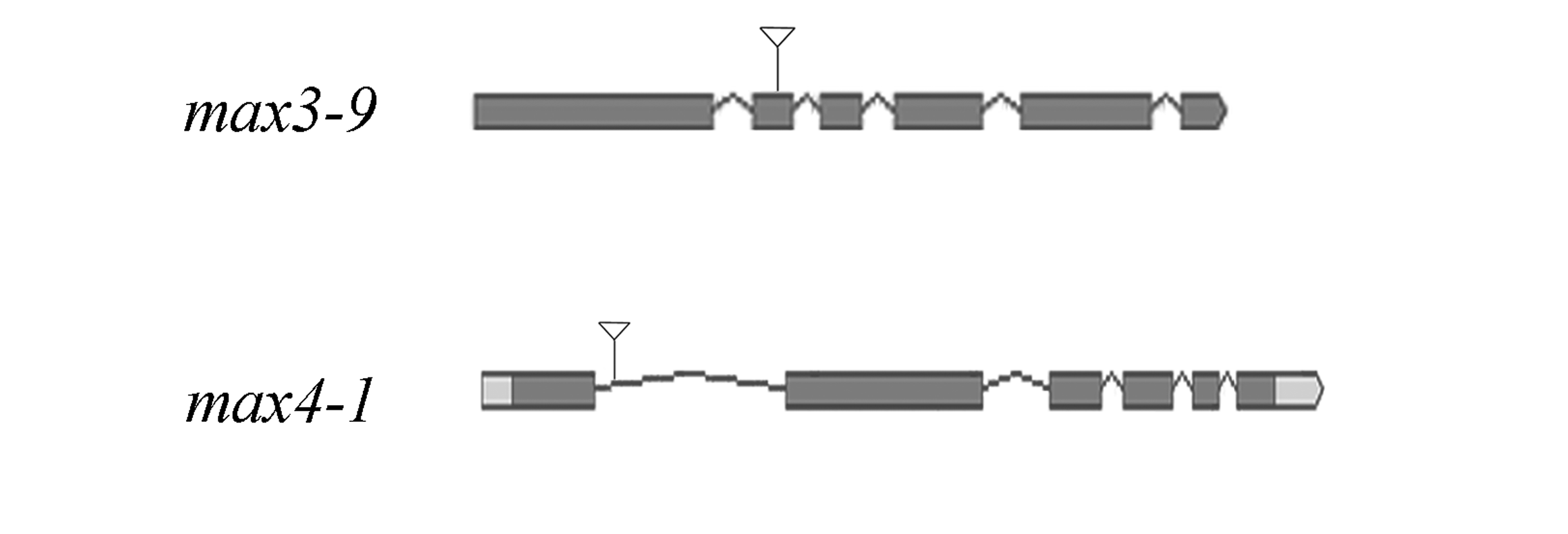

Supplement: Additional file 1 — Schematic diagram of disrupted carotenoid cleavage dioxygenase genes CCD7 and CCD8 in the max3-9 and max4-1 mutants used in this study. Boxes represent exons and lines represent introns. Triangles show the T-DNA insertion sites. The max mutants were previously reported by (Booker et al. 2004). [file 1471-2229-12-169-S1.png]

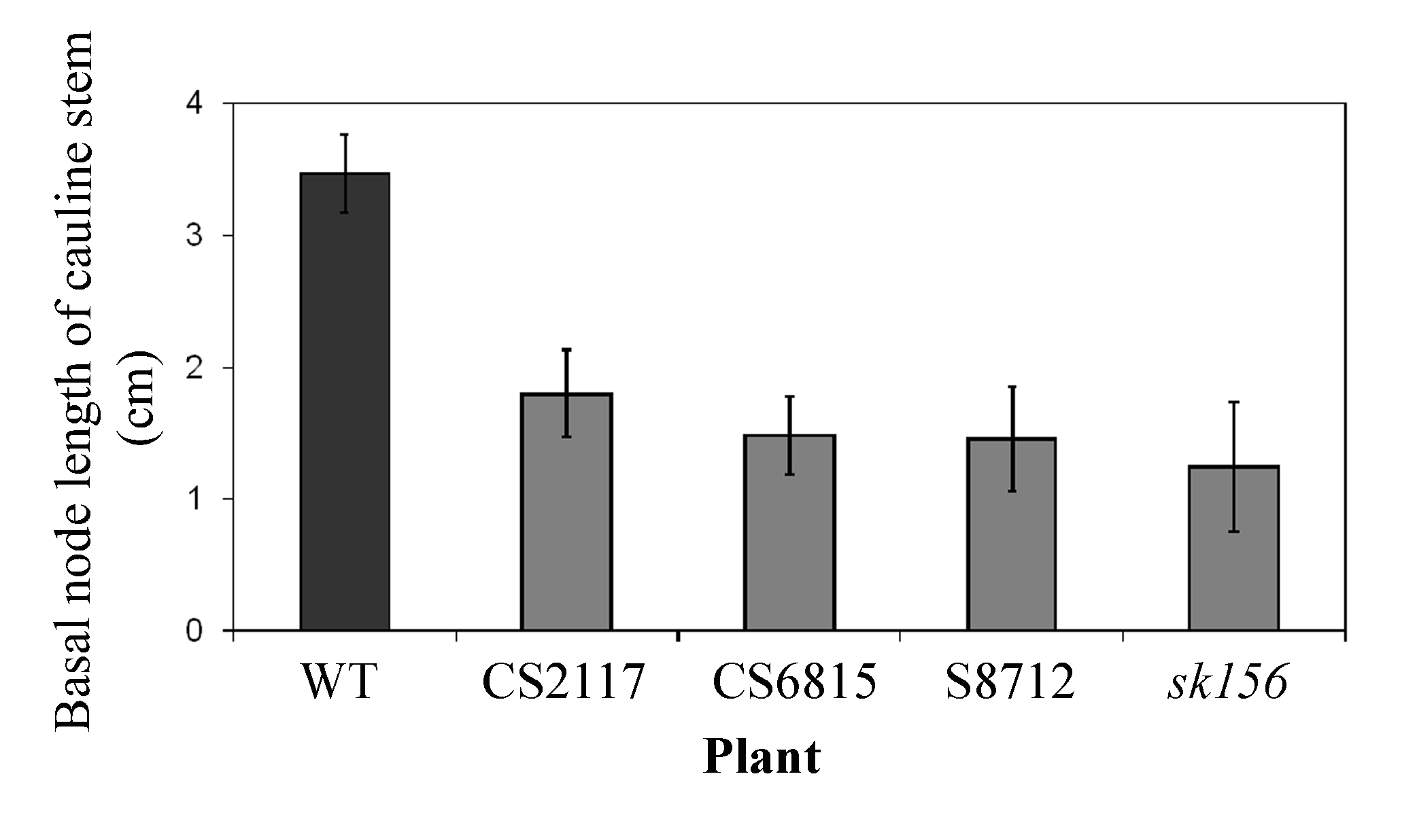

Supplement: Additional file 3 — Reduced lengths of cauline stem basal internodes in three spl15 mutants compared with WT Arabidopsis. Length of the cauline stem basal inter-node was measured from the rosette core up to the first visible basal node for WT, three spl mutants and sk156 plants grown for 6 weeks. [file 1471-2229-12-169-S3.png]

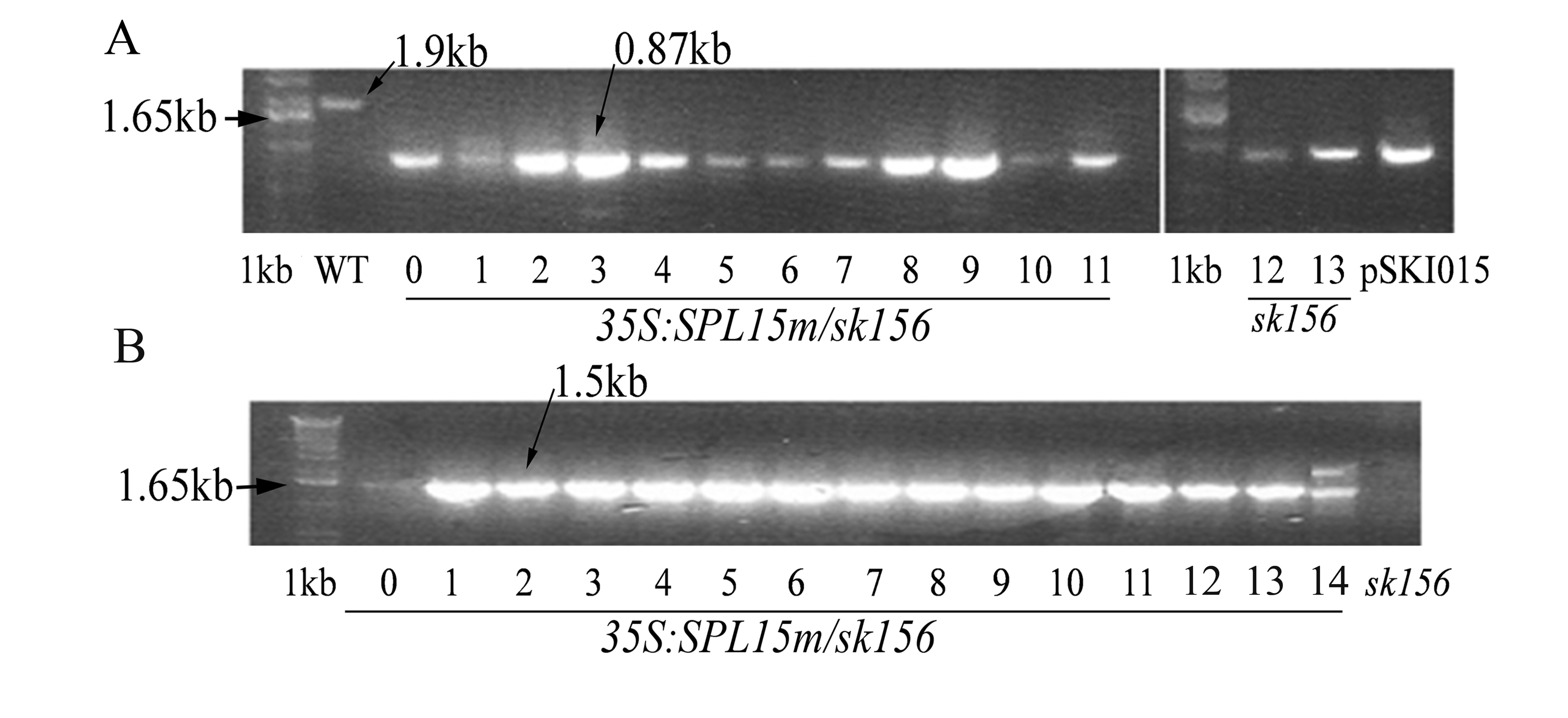

Supplement: Additional file 4 — Confirmation by PCR of transgene presence in sk156 lines transformed with a 35S:SPL15m gene. The miR156 insensitive SPL15m contained 11 mutated nucleotides as described in Materials and Methods. Primer sequences are listed in Additional file 5. (A) Activation-tag from pSKI015 T-DNA detected in transgenic sk156 plants carrying 35S:SPL15m cassette (lanes 0-11) and in the sk156 background alone (lanes 12 and 13). Primers SK2222-F (430bp upstream) and SK2222-R (1830bp downstream) flanking the T-DNA insertion site and primer pSKI015-GW-LB2 (439bp to the T-DNA left border) were used to detect the insert. In WT lane, no T-DNA insert was detected and only a fragment close to 1.9kb was present due to genomic DNA amplified with the primers flanking the T-DNA insertion site. In homozygous sk156 plants which did not carry 35S:SPL15m, a single T-DNA fragment (869bp) was generated. 1kb, 1-kb Plus DNA ladder (Invitrogen); WT, Col-4; pSKI015, plasmid containing the activation tag present in sk156. (B) Transgene SPL15m detected in the transgenic sk156 lines carrying 35S:SPL15m (lanes 0-14), but not in sk156 alone using primers 35SF3 and SPL15R. [file 1471-2229-12-169-S4.png]

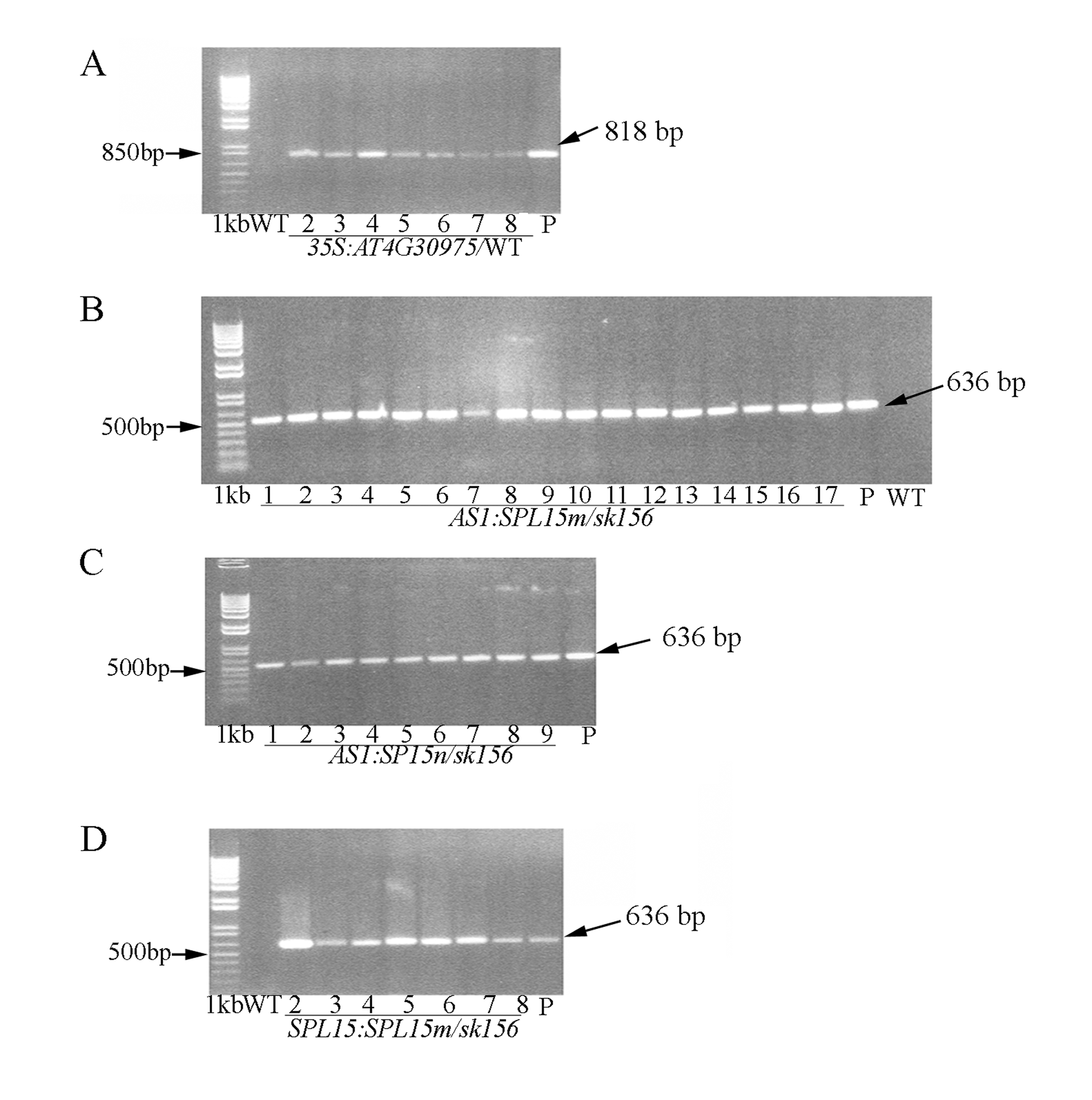

Supplement: Additional file 6 — PCR confirmation of transgene presence in four different miR156-sensitive or miR156-insensitive transgenic Arabidopsis populations used in this manuscript. (A) Lanes 2-8, PCR product (818bp) for 7 transgenic plants carrying a 35S:AT4G30975 cassette in a WT background using primers 35S-F3 and p795-3R. P, binary plasmid pBI121 containing 35S:AT4G30975 as a positive control. WT, Col-4. (B), (C) and (D) Transgene PCR product (636bp) carrying AS1:SPL15m, AS1:SPL15n and SPL:SPL15m cassettes in a sk156 background, respectively, using primers SPL15-871F and NosTer-R6. P, plasmid containing 35S:SPL15 as a positive control. Black arrows points to the DNA marker. Primer sequences are listed in Additional file 5. 1kb, 1-kb Plus DNA ladder (Invitrogen). [file 1471-2229-12-169-S6.png]
