# Supplementary material for: Arabidopsis mutant sk156 reveals complex regulation of SPL15 in a miR156-controlled gene network
Source: BMC Plant Biol. 2012 Sep 18;12:169. doi: 10.1186/1471-2229-12-169 (PMC3520712; doi:10.1186/1471-2229-12-169)
Supplement: Additional file 2 — SALK and FLAG T-DNA insertion lines for miR156-targeted SPL genes. [file 1471-2229-12-169-S2.doc]

|  | | |  |  |
| --- | --- | --- | --- | --- |
|  | | | | |
|  |  |  |  |  |
| **SPL gene** | **Accession#** | **Mutant** | **Insertion site** | **Stock Number** |
| SPL 2 | AT5G43270 | FLAG_245C09 | exon | EBNTV100T3 |
|  |  | SALK_022235 | intron | SALK_022235 |
| SPL 3 | AT2G33810 | SALK_035917 | promoter | SALK_035917 |
|  |  | FLAG_173C12 | exon | DUSTV10T3 |
| SPL 4 | AT1G53160 | SALK_137581 | 3'UTR | SALK_137581 |
| SPL 6 | AT1G69170 | SALK_047690 | promoter | SALK_047690 |
| SPL 9 | AT2G42200 | SAIL_420_D11 | Promoter | CS819406 |
| SPL 10 | AT1G27370 | FLAG_295G12 | exon | COGTV38T3 |
|  |  | SALK_112209 | 5UTR | SALK_112209 |
| SPL 11 | AT1G27360 | WiscDsLox481-484A20 | promoter | CS857902 |
|  |  | SALK_122018 | far upstream promoter | SALK_122018 |
| SPL 13 | AT5G50570 | SALK_104630 | promoter, -73bp | SALK_104630 |
| SPL 15 | AT3G57920 | WiscDsLox345-348I4 | promoter | CS852117 |
|  |  | WiscDsLox457-460B23 | exon | CS856815 |
|  |  | SALK_138712 | intron | SALK_138712 |
