# Supplementary material for: Arabidopsis mutant sk156 reveals complex regulation of SPL15 in a miR156-controlled gene network
Source: BMC Plant Biol. 2012 Sep 18;12:169. doi: 10.1186/1471-2229-12-169 (PMC3520712; doi:10.1186/1471-2229-12-169)
Supplement: Additional file 5 — Primers used in this study. [file 1471-2229-12-169-S5.doc]

|  | | | |  |  | |  |  |
| --- | --- | --- | --- | --- | --- | --- | --- | --- |
|  | | | | | | | |  |
|  |  | | |  |  | | |  |
| **Primer name** | **Gene** | **Locus #** | **Primer sequence** | | | **Usage** | |  |
| pbHLH_F | bHLH069 | AT4G30980 | 5’ tctagaaacattcattttcattctcactccc 3’ | | | Gene cloning | |  |
| pbHLH_R | bHLH069 | AT4G30980 | 5’gagctcttaaggatataactttataaaccaaa 3’ | | | Gene cloning | |  |
| miR156b_XbaI_F | miR156b | AT4G30972 | 5’ tctagagtaagacacgtgtagaaatcttc 3’ | | | Gene cloning | |  |
| miR156b_SacI_R | miR156b | AT4G30972 | 5’gagctctcagggtgaagcacattag 3’ | | | Gene cloning | |  |
| p30975BamHI_F | Part of miR156b | AT3G30975 | 5' aaaggattcgcttgacctctctctctctctctc 3' | | | Gene cloning | |  |
| p30975SacI_R | Part of miR156b | AT3G30975 | 5' aaa agagctctaagcttggttacaggttctagc 3' | | | Gene cloning | |  |
| pXbaI _SPL15F | SPL15 | AT3G57920 | 5’ tctagactctctcttctcttctctctgatt 3’ | | | Gene cloning | |  |
| pSacI _SPL15R | SPL15 | AT3G57920 | 5’ gagctcgaaaaaaacatccaaacggaaagg 3’ | | | Gene cloning | |  |
| pSPL15m851F | SPL15m | AT3G57920 | 5’ aagcttgttaagcaactcatacccaattcatcagc 3’ | | | SPL15 mutation | |  |
| pSPL15m865R | SPL15m | AT3G57920 | 5’gcttaacaagcttagagcacagcttgaatctgtg 3’ | | | SPL15 mutation | |  |
| pAS1_HindIII_F | AS1 promoter | AT2G37630 | 5'aaaaagcttacggagggtgtgagtgagtagtggta 3' | | | promoter cloning | |  |
| pAS1_XbaI_R | AS1 promoter | AT2G37630 | 5' aaatctagactcctactcctcctgacatcacttct 3' | | | promoter cloning | |  |
| pSPL15Pr3_HindIII_F | SPL15 promoter | AT3G57920 | 5'aaaaaagcttgatattgctcttccctcctagtttcc 3' | | | promoter cloning | |  |
| pSPL15Pr3_XbaI_R | SPL15 promoter | AT3G57920 | 5' aaaatctagaaaagaaagaaagagaagctgac 3' | | | promoter cloning | |  |
| p15SBP-BamHI-F | SPL15 | AT3G57920 | 5' aaaaggatccaccgttcgtaagtcgtctaccacg 3' | | | SBP expression | |  |
| p15SBP-SalI-R | SPL15 | AT3G57920 | 5'aaaagtcgactcagaaaagagccgttgtgggttgtgg3' | | | SBP expression | |  |
| R156b-bx1-U | miR156 promoter | AT3G30975 | 5'taattagtgaaactgtacaatgtacaatgtaccaatgccattaatg3' | | | SBP binding DNA | |  |
| R156b-bx1-L | miR156 promoter | AT3G30975 | 5'cattaatggcattggtacattgtacattgtacagtttcactaatta 3' | | | SBP binding DNA | |  |
| SK2222-F | Flanking T-DNA insertion site | AT4G30980 | 5' cgcaacctcagacaaagcctaaagt 3' | | | Homozygocity | |  |
| SK2222-R | Flanking T-DNA insertion site | AT4G30980 | 5' agaagcagcaccgcccagtctactc 3' | | | Homozygocity | |  |
| pSKI015-GW-LB1 | T-DNA | pSKI015 | 5' gtgggccccaaatgaagtgcaggtcaaac 3' | | | Homozygocity | |  |
| pSKI015-GW-LB2 | T-DNA | pSKI015 | gttgggcgggtccagggcgaattttgc | | | Homozygocity | |  |
| qPCRat4g30960_603F | Adjacent gene | AT4G30960 | 5' gagcttcacgaagtcatggcgag 3' | | | qPCR | |  |
| qPCRat4g30960_748R | Adjacent gene | AT4G30960 | 5' acggctgagatcaattgctggaaa 3' | | | qPCR | |  |
| qPCRat4g30970_117F | Adjacent gene | AT4G30970 | 5' ggtggctacttggctgcaaaatga 3' | | | qPCR | |  |
| qPCRat4g30970_266R | Adjacent gene | AT4G30970 | 5' ttgttggttactggcggattgaca 3' | | | qPCR | |  |
| qPCRbHLH_730F | bHLH069 | AT4G30980 | 5' acggagcaccaagttgcaaagcta 3' | | | qPCR | |  |
| qPCRbHLH_872R | bHLH069 | AT4G30980 | 5' acgaaggggctacgagaaggacac 3' | | | qPCR | |  |
| qSPL15_1142R | SPL15 | AT3G57920 | 5’ ggaaatctgctggctccgaga 3’ | | | qPCR | |  |
| qSPL15_993F | SPL15 | AT3G57920 | 5’ cagccaccgcccatttcaac 3’ | | | qPCR | |  |
| qPCRmiR156_12F | miR156b | AT4G30972 | 5' gagagatggtgattgaggaatgc 3' | | | qPCR | |  |
| qPCRmiR156_140R | miR156b | AT4G30972 | 5' cagagataggcaactgacagaaagag 3' | | | qPCR | |  |
| qAT5G25760_383F | reference gene | AT5G25760 | 5' tgcttggagtcctgcttgga 3' | | | qPCR | |  |
| qAT5G25760_529R | reference gene | AT5G25760 | 5' tgtgccattgaattgaaccctct 3' | | | qPCR | |  |

(To be continued)

| **Primer name** | **Gene** | **Locus #** | **Primer sequence** | **Usage** |
| --- | --- | --- | --- | --- |
| pSPL15-3R | SPL15 | AT3G57920 | 5' gaaaaaaacatccaaacggaaaggc 3' | TDNA detection |
| 35S-F3 | CaMV promoter | pBI121 | 5' caatcccactatccttcgcaagaccc 3' | TDNA detection |
| p975-3R | Part of miR156b | AT4G30975 | 5' gctagaacctgtaaccaagctta 3' | TDNA detection |
| pSPL15-871F | SPL15 | AT3G57920 | 5' ttcgactcgatgatctcattctcc 3' | TDNA detection |
| NosTer-R6 | Nos terminator | pBI121 | gatctagtaacatagatgacaccg | TDNA detection |
| SPL2_22235R | SPL 2 | AT5G43270 | 5' att tct tct tcc tct ggg atc cat aag 3' | TDNA detection |
| SPL2_22235F | SPL 2 | AT5G43270 | 5' cccattgaaacggtggctttgcatta 3' | TDNA detection |
| SPL2_FLAG_R | SPL 2 | AT5G43270 | 5' ctctaaaagctcaaagtcaacttcc 3' | TDNA detection |
| SPL2_FLAG_F | SPL 2 | AT5G43270 | 5' actaaatgctataactgaactatagg 3' | TDNA detection |
| SPL3_5917F | SPL 3 | AT2G33810 | 5' gtttggaccattcttgtatatcc 3' | TDNA detection |
| SPL3_5917R | SPL 3 | AT2G33810 | 5' gcatgttgtgtaaaaaagagtacgaac 3' | TDNA detection |
| SPL3_FLAG_F | SPL 3 | AT2G33810 | 5' gaa gaa gaa gag gct ttg gag aag 3' | TDNA detection |
| SPL3_FLAG_R | SPL 3 | AT2G33810 | 5' cag aac tac ata tat cac gaa tc 3' | TDNA detection |
| SPL4_7581F | SPL 4 | AT1G53160 | 5' ctctcaggacttaaccaacgc 3' | TDNA detection |
| SPL4_7581R | SPL 4 | AT1G53160 | 5' gatgacgtctaaggagtctc 3' | TDNA detection |
| SPL6_7690F | SPL 6 | AT1G69170 | 5' cgctttggacgatgttcattg 3' | TDNA detection |
| SPL6_7690R | SPL 6 | AT1G69170 | 5' gaggatcgaagaataagacttaccg 3' | TDNA detection |
| SPL9_9460F | SPL 9 | AT2G42200 | 5' catttttgagttatatttctttac 3' | TDNA detection |
| SPL9_9460R | SPL 9 | AT2G42200 | 5' ccaaacatgagccctccactgaaagag 3' | TDNA detection |
| SPL10_2209F | SPL 10 | AT1G27370 | 5' ctctcttctcttactttactcacc 3' | TDNA detection |
| SPL10_2209R | SPL 10 | AT1G27370 | 5' gaattccaagcaccacacactgc 3' | TDNA detection |
| SPL11_7902F | SPL 11 | AT1G27360 | 5' ggtgaatggtctctaggtatag 3' | TDNA detection |
| SPL11_7902R | SPL 11 | AT1G27360 | 5' cattcacgatatgggttgatttcatag 3' | TDNA detection |
| SPL13_4630F | SPL 13 | AT5G50570 | 5' ctccctgataattgacaacttgtg 3' | TDNA detection |
| SPL13_4630R | SPL 13 | AT5G50570 | 5' cattaaaaaaaactatagaataagaaaag 3' | TDNA detection |
| SPL15_2117F | SPL 15 | AT3G57920 | 5' gcctgacccgaacacattaaca 3' | TDNA detection |
| SPL15_2117F | SPL 15 | AT3G57920 | 5' gggggctttaggccttttt 3' | TDNA detection |
| SPL15_6815R | SPL 15 | AT3G57920 | 5' ttgcagcattggggtttcct 3' | TDNA detection |
| SPL15_6815R | SPL 15 | AT3G57920 | 5' accaccaaaacgcaccaaca 3' | TDNA detection |
| SPL15_8712F | SPL 15 | AT3G57920 | 5' ttgcagcattggggtttcct 3' | TDNA detection |
| SPL15_8712R | SPL 15 | AT3G57920 | 5'accaccaaaacgcaccaaca 3' | TDNA detection |
| LBb1 | T-DNA | pROK2 vector | 5' gcgtggaccgcttgctgca 3' | TDNA detection |
| p745 | T-DNA | pDs-Lox | 5' aacgtccgcaatgtgttattaagttgtc 3' | TDNA detection |

(Continued for Supplementary table 1)
